# Molecular genetic characteristics and clinical significance of childhood acute lymphoblastic leukemia

**DOI:** 10.3389/fped.2025.1665431

**Published:** 2025-10-21

**Authors:** Zhenzhen Zhu, Junchen Yan

**Affiliations:** ^1^ Blood Donation Service Department, Wuhan Blood Center, Wuhan, China; ^2^ General Internal Medicine Department, Wuhan Children's Hospital, Wuhan, China

**Keywords:** childhood acute lymphoblastic leukemia, molecular genetics, fusiongene, minimal residual disease, prognosis

## Abstract

**Objective:**

To explore the molecular genetic characteristics of childhood acute lymphoblastic leukemia (ALL) and their relationships with clinical phenotypes, chromosomal abnormalities, and prognosis, so as to provide references for precise diagnosis and treatment.

**Methods:**

A total of 302 newly diagnosed children with ALL were included. Real-time fluorescent quantitative PCR, high-throughput sequencing and other technologies were used to detect common fusion genes, rare fusion genes and Ph-like ALL-related molecules. Combined with chromosomal karyotype analysis, immunophenotyping and minimal residual disease (MRD) monitoring, the associations between molecular genetic characteristics and clinical indicators as well as prognosis were analyzed.

**Results:**

Among the 302 children, the total positive rate of leukemia genes was 50.66%, and the gene detection rate in B-ALL children (52.90%) was significantly higher than that in T-ALL (37.21%). Common fusion genes were mainly ETV6/RUNX1 (19.54%), MLL (9.27%) and BCR/ABL (6.29%), with age- and immune subtype-specific distributions. Children with ETV6/RUNX1 positivity had the highest complete remission rate (93.2%) and the best 2-year event-free survival rate (89.8%), while those with BCR/ABL positivity had the worst prognosis (complete remission rate 57.9%, 2-year event-free survival rate 42.1%). There were differences in the consistency between fusion genes and chromosomal abnormalities: ETV6/RUNX1 and BCR/ABL showed 100% consistency with specific chromosomal translocations, while E2A/PBX1 and MLL showed about 50% consistency. The dynamic changes of MRD were closely related to gene types, with significantly higher MRD positive rates in children with high-risk genes.

**Conclusion:**

The molecular genetic characteristics of childhood ALL have clear clinical significance. Fusion gene detection can be used for disease classification, risk stratification and prognosis evaluation, providing an important basis for the formulation of individualized treatment strategies.

## 1 Introduction

Childhood acute lymphoblastic leukemia (ALL) is the most common malignant tumor in childhood, accounting for 70%–80% of childhood leukemias (1). With the progress of diagnosis and treatment technologies, the overall cure rate of childhood ALL has improved significantly. However, the prognosis varies greatly among different children. Some high-risk subtypes still face treatment challenges due to drug resistance, recurrence and other issues (2). Molecular genetic changes, especially the formation of fusion genes, are considered the core mechanism driving the occurrence and development of ALL (3, 4). They play a key role in disease classification, risk assessment and treatment selection (5). At present, although studies have revealed the clinical significance of some common fusion genes such as ETV6/RUNX1 and BCR/ABL, there is still a lack of systematic and in-depth research on Chinese children regarding the distribution characteristics of rare fusion genes, Ph-like ALL-related molecular abnormalities, and their associations with epigenetic modifications and dynamic changes of minimal residual disease (MRD). These gaps limit the further optimization of precise diagnosis and treatment (6, 7).

The innovations of this study are mainly reflected in three aspects. First, in a cohort of 302 newly diagnosed Chinese children with ALL, it is the first time to comprehensively integrate detection data of common fusion genes, rare fusion genes such as ZNF384 rearrangement and DUX4 rearrangement, and Ph-like ALL-related molecules such as CRLF2 rearrangement and JAK2 mutation. It clarifies the specific distribution patterns of different molecular genetic characteristics in different age groups and immune subtypes, making up for the lack of previous studies on rare genes and Ph-like subtypes in Chinese children. Second, it innovatively combines fusion gene detection with epigenetic modification analysis such as ATP-binding cassette subfamily B member 1 (ABCB1) and TP53 promoter methylation and H3K4me3 modification. It reveals the synergistic role of epigenetic regulation in fusion gene-driven leukemia occurrence and chemotherapy resistance. It also verifies the targeted inhibitory effect of decitabine on MLL-positive cells through *in vitro* experiments, providing experimental basis for combined epigenetic drug therapy. Third, it systematically analyzes the association between fusion gene types and dynamic changes of MRD, especially the impact of co-mutations such as BCR/ABL combined with FLT3/ITD on MRD clearance rate. It combines static genetic characteristics with dynamic treatment responses to improve the accuracy of risk stratification.

The value of this study lies in systematically analyzing the molecular genetic characteristics of childhood ALL and their associations with clinical phenotypes, treatment responses and prognosis. It provides a more comprehensive basis for precise disease classification and risk stratification, and opens up new directions for formulating individualized treatment strategies. For example, it supports the application of JAK-STAT pathway inhibitors for Ph-like ALL and the combination of demethylating drugs and chemotherapy for MLL-positive children. Meanwhile, the results emphasize the importance of combining fusion gene detection with dynamic MRD monitoring. This helps identify high-risk children earlier and adjust treatment plans in time, ultimately laying a foundation for improving the overall efficacy of childhood ALL and the long-term prognosis of children.

## 2 Subjects and methods

### 2.1 Study subjects

This study selected 302 newly diagnosed children with ALL from Wuhan Children's Hospital as subjects. All children met the diagnostic criteria specified in guidelines for the diagnosis and treatment of childhood ALL and had complete clinical data (8, 9). Children with other malignant tumors, congenital immunodeficiency diseases, or severe organ dysfunction were excluded to ensure the homogeneity of subjects and the reliability of data. The study was approved by the Ethics Committee of Wuhan Children's Hospital, and written informed consent was obtained from the guardians of all participating children.

### 2.2 Main experimental instruments and reagents

The main instruments used in the experiment included: flow cytometer (BD FACSCanto II, BD Company, USA) for immunophenotyping; real-time fluorescent quantitative PCR instrument (Roche LightCycler 480, Roche Company, Switzerland) for fusion gene detection; chromosome karyotyping system (CytoVision, Leica Company, USA) for chromosome karyotype analysis; and automatic hematology analyzer (Sysmex XN-9000, Sysmex Company, Japan) for blood routine testing. All instruments underwent strict calibration and quality control to ensure the accuracy of test results.

Immunophenotyping monoclonal antibodies, including CD19, CD20, CD10, CD3, CD4, and CD8, were purchased from BD Company (USA). Fusion gene detection kits, which cover ETV6/RUNX1, E2A/PBX1, MLL, and BCR/ABL, were provided by Da An Gene Co., Ltd. (China). For cell culture, RPMI 1,640 medium and fetal bovine serum were obtained from Gibco Company (USA). Colchicine, KCl hypotonic solution, and methanol-glacial acetic acid fixative (at a ratio of 3:1) used for chromosome preparation were sourced from Sigma Company (China). All reagents were utilized within their expiration dates and handled strictly in accordance with the manufacturer's instructions.

### 2.3 Experimental methods

#### 2.3.1 Collection of clinical data and blood routine testing

Clinical data of the children were collected, including gender, age, and blood routine indicators at initial diagnosis [white blood cell count (WBC), hemoglobin (Hb), platelet count (PLT)]. Blood routine indicators were detected using an automatic hematology analyzer. Strictly following the instrument operation procedures, each sample was tested twice. The average value was taken as the final result to reduce detection errors.

#### 2.3.2 Immunophenotyping detection

Bone marrow or peripheral blood samples (2–3 ml) from the children were collected. After anticoagulation with EDTA, mononuclear cells were separated by density gradient centrifugation at 2,000 r/min for 15 min. The cell concentration was adjusted to 1 × 10⁶ cells per ml. Fluorescently labeled monoclonal antibodies such as CD19-PE, CD10-FITC and CD3-APC were added. The mixture was incubated at 4°C in the dark for 30 min. Then, the cells were washed twice with PBS and analyzed by flow cytometry. Based on the expression patterns of cell surface antigens, ALL was classified into B-ALL and T-ALL. B-ALL was further subdivided into pro-B-ALL, pre-B-ALL and C-B-ALL. The classification criteria were referenced from guidelines for immunophenotypic analysis of acute leukemia by flow cytometry (10).

#### 2.3.3 Fusion gene detection

Fusion genes were detected by real-time fluorescent quantitative PCR. First, bone marrow or peripheral blood samples were taken, and genomic DNA (gDNA) was extracted using the magnetic bead method. The extraction process was strictly performed according to the kit instructions. DNA concentration and purity were measured, with the A260/A280 ratio required to be between 1.8 and 2.0 to ensure gDNA integrity. The PCR reaction system consisted of 10 μl of 2 × PCR Mix (containing DNA polymerase, dNTPs, and fluorescent probes), 0.5 μl each of gene-specific forward and reverse primers (targeting fusion breakpoints in genomic DNA), 2 μl of gDNA template (50–100 ng/μl), and nuclease-free ddH₂O to make up to 20 μl.

Quality controls were included in each qPCR run: (1) Positive control: Plasmid containing the target fusion gene sequence (to verify reaction efficiency); (2) Negative control: gDNA from healthy donors (to exclude non-specific amplification); (3) No-template control (NTC): Nuclease-free ddH_2_O (to detect reagent contamination).

The reaction conditions were as follows: pre-denaturation at 95 °C for 5 min; 40 cycles of denaturation at 95 °C for 15 s, annealing at 60 °C for 30 s, and extension at 72 °C for 30 s; and final extension at 72 °C for 10 min. Results were judged based on the fluorescence curve. A Ct value < 38 was considered positive (fusion gene present in genomic DNA), and ≥38 was negative. Positive samples needed to be re-verified using Sanger sequencing of the PCR product to confirm the fusion breakpoint and ensure the reliability of results.

#### 2.3.4 Detection of rare fusion genes and Ph-like ALL-related molecules

All children underwent deep whole-exome sequencing (WES, based on genomic DNA) via high-throughput technology on the Illumina NovaSeq 6,000 platform (Illumina, USA), with the detection scope covering Ph-like acute lymphoblastic leukemia (ALL)-related genes (CRLF2, JAK2, IL7R, SH2B3), rare fusion genes (e.g., ZNF384 rearrangement, DUX4 rearrangement), and other low-frequency mutations (FLT3/ITD, NRAS, KRAS).

Genomic DNA was first fragmented into 150–200 bp segments using a sonication device to optimize fragment size for subsequent exome capture. Exome enrichment and genomic library construction were performed using the Agilent SureSelect Human All Exon V6 kit (Agilent Technologies, USA): this kit contains biotin-labeled oligonucleotide probes that specifically hybridize to human exonic regions. After hybridization, streptavidin-coated magnetic beads were added to selectively bind biotin-labeled probe-exonic fragment complexes, enabling efficient enrichment of targeted exonic sequences while removing non-exonic DNA (e.g., intronic and intergenic regions). The enriched exonic fragments were then amplified via PCR to generate sufficient DNA, forming the final exome library that met high-throughput sequencing requirements.

Sequencing was conducted using a paired-end strategy with a read length of 150 bp × 2—this setup enhances the accuracy of identifying sequence variations and fusion gene breakpoints. To ensure reliable detection of low-frequency mutations (allele frequency ≥5%) in leukemia cell subclones, the average sequencing depth was maintained at ≥100×, which guarantees each base in exonic regions has adequate read coverage to distinguish true mutations from technical errors.

A standardized bioinformatics pipeline (adapted for DNA-based WES data) was applied for sequencing data processing and analysis, as follows:

Raw Data Quality Control: FastQC software was used to assess the quality of raw sequencing reads. Reads were filtered out if they met either of the following criteria: a Phred quality score (Q30) of <80% (indicating low base-calling accuracy) or detectable adapter contamination (resulting from residual sequencing adapters not removed during library preparation).

Read Alignment: The filtered high-quality (“clean”) reads were aligned to the human reference genome (GRCh38/hg38 assembly) using the BWA-MEM algorithm, a tool optimized for accurate alignment of short sequencing reads. Reads with a mapping quality (MAPQ) score <20 were discarded, as low MAPQ scores indicate uncertainty about the correct genomic location of the read, which could lead to false-positive variant calls.

Given that WES data (DNA-based) primarily covers exonic regions, fusion genes were identified using two complementary DNA-specific structural variation (SV) calling tools to improve detection accuracy. The first tool was Delly 0.9.1, which is optimized for detecting balanced translocations, deletions, and insertions from paired-end WES data; it identifies potential fusion breakpoints by analyzing abnormal read pairs (e.g., read pairs mapping to different chromosomes) and split reads (reads spanning breakpoint junctions). The second tool was Manta 1.6.0, a high-sensitivity SV caller that integrates signals from paired-end reads, split reads, and read depth changes to detect fusion events—this tool is particularly effective for identifying fusion events with breakpoints in exonic or near-exonic regions, which aligns with the coverage scope of WES data and ensures that potential fusion genes relevant to the study (e.g., ZNF384 rearrangement, DUX4 rearrangement) are not missed due to breakpoint location limitations.

To reduce false positives caused by technical errors or non-pathogenic genetic variations in WES data (DNA-based), only fusion events meeting strict filtering criteria were retained for subsequent analysis. Specifically, the retained fusion events must first be supported by ≥8 independent split reads (a higher threshold than the initial ≥5, adjusted to adapt to the characteristics of WES data that primarily covers exonic regions and has limited intronic coverage) and/or ≥10 abnormal read pairs, ensuring sufficient sequencing read support for true fusion breakpoints. Second, their fusion breakpoints must be located within exonic regions or flanking intronic regions (≤100 bp from exons) covered by the Agilent SureSelect V6 probe set, excluding fusion events with breakpoints in non-targeted regions that exceed the detection scope of WES. Third, the fusion events must not match any known non-pathogenic fusion entries in the Database of Genomic Variants, further filtering out benign genetic variations that are irrelevant to childhood ALL.

Orthogonal Validation of Fusion Genes: All candidate fusion genes (e.g., ZNF384 rearrangement, DUX4 rearrangement, NUP214/ABL1 fusion) identified from WES data were validated using two orthogonal methods to ensure the reliability of detection results.

Fluorescence *in situ* Hybridization (FISH): Specific FISH probes targeting the fusion partner genes (e.g., ZNF384, DUX4, NUP214, ABL1) were designed and synthesized (Empire Genomics, USA). Bone marrow smears were hybridized with FISH probes, and signals were observed under a fluorescence microscope (Leica DM6 B, Germany). Fusion events were confirmed by the presence of rearranged signals (e.g., split signals, colocalized signals) in ≥10% of analyzed cells.

Sanger Sequencing: PCR primers were designed to amplify the fusion breakpoint region (based on WES-identified breakpoint coordinates). Genomic DNA from positive samples was used as a template for PCR amplification, and the PCR products were sequenced using the ABI 3730xl Genetic Analyzer (Thermo Fisher Scientific, USA). The obtained sequences were aligned to the human reference genome (GRCh38/hg38) to confirm the exact fusion breakpoint and partner gene.

Low-Frequency Mutation Detection: GATK 4.0 software was employed to detect low-frequency mutations (e.g., JAK2 mutations, IL7R mutations). Mutations were filtered using strict criteria: a variant quality score (QUAL) ≥ 30 (reflecting high confidence in the variant call), a coverage depth ≥10× (ensuring sufficient read support), and an allele frequency ≥5% (consistent with the study's focus on clinically relevant subclonal mutations).

Ph-like ALL Diagnostic Algorithm and Confirmatory Assays: The diagnostic workflow consisted of three sequential steps. First, in the screening for common fusion gene exclusion, all cases were tested for common fusion genes using RT-qPCR and Sanger sequencing. Cases positive for any of these common fusion genes were excluded from Ph-like ALL classification, and only fusion gene-negative cases were further subjected to Ph-like ALL-related testing. Second, for the detection of Ph-like core lesions, fusion gene-negative cases underwent targeted detection of Ph-like-related abnormalities through a combination of assays. For CRLF2 rearrangement, two subtypes were distinguished: via Fluorescence *in situ* Hybridization (FISH), dual-color break-apart probes for CRLF2 (Empire Genomics, USA) and fusion probes for IGH-CRLF2 [corresponding to chromosomal translocation *t*(14; 19) (q32; p13.2)] and P2RY8-CRLF2 (resulting from intrachromosomal deletion at 19p13.2) were used, with signals counted in no fewer than 200 interphase cells-cases with ≥10% abnormal cells were defined as positive; via RT-PCR, specific primers targeting the junction regions of IGH-CRLF2 (forward: 5′-GAGGGAAGGGGAAGACATTT-3′, reverse: 5′-CTTGGGTGGTTTTGGTGTTG-3′) and P2RY8-CRLF2 (forward: 5′-TGGCTACAGTGTCCTGGTTC-3′, reverse: 5′-CAGCAGCAGACACAGAGTCA-3′) were adopted, and a Ct value < 38 was defined as positive (55). For JAK/IL7R pathway mutations, mutations in JAK2 (exons 12–14), IL7R (exons 6–8), and SH2B3 (exons 2–4) were detected using deep whole-exome sequencing (WES, Illumina NovaSeq 6,000) with an average sequencing depth of ≥200×; variants were filtered using GATK 4.0 software (with a variant quality score [QUAL] ≥ 50 and an allele frequency ≥5%) and annotated via ANNOVAR software, and their pathogenicity was confirmed by matching with the COSMIC database. For ABL-class fusions, rare fusions (e.g., NUP214/ABL1, ETV6/ABL1) were identified via WES (using Delly 0.9.1 and Manta 1.6.0 software for structural variation calling) and validated through FISH (with ABL1 break-apart probes) and Sanger sequencing. Third, in the confirmatory testing for the “pure Ph-like” subtype, cases were labeled as “pure Ph-like” if they met two criteria: no common fusion genes were detected (verified via RT-qPCR and Sanger sequencing), and at least one Ph-like core lesion was confirmed by two orthogonal assays. Quality controls were implemented for each assay to ensure reliability: for FISH, a positive control (CRLF2-rearranged cell line MUTZ-5) and a negative control (healthy donor bone marrow) were included; for RT-PCR, a positive control (plasmids containing IGH-CRLF2/P2RY8-CRLF2 sequences) and a no-template control (nuclease-free water) were used; for WES, a reference standard (NA12878 cell line DNA) was employed to guarantee the accuracy of variant calling.

Variant Annotation: All detected variants (including mutations and fusion genes) were annotated using ANNOVAR software. This tool integrates information from multiple public databases, including dbSNP (for known single-nucleotide polymorphisms), ExAC (for population frequency data), and COSMIC (for cancer-related variant annotations). Annotation results included details such as variant type (e.g., missense mutation, frameshift insertion/deletion), predicted pathogenicity, and associations with known diseases, which facilitated the identification of functionally relevant variants linked to childhood acute lymphoblastic leukemia.

#### 2.3.5 Chromosome karyotype analysis

The steps for bone marrow cell culture were as follows. 1–2 ml of bone marrow samples were inoculated into RPMI 1,640 medium containing 10% fetal bovine serum. They were cultured in a 37 °C, 5% CO₂ incubator for 72 h. Colchicine was added 2 h before the end of culture to a final concentration of 0.1 μg/ml to stop cell division. After harvesting cells, they were treated with 0.075 mol/L KCl hypotonic solution for 30 min, fixed three times with methanol-glacial acetic acid at a ratio of 3:1, dropped onto slides, air-dried naturally, and stained with Giemsa. Karyotype analysis was performed using a chromosome karyotyping system. Twenty metaphase spreads were analyzed for each case. Karyotype description referred to International System for Human Cytogenomic Nomenclature (ISCN 2020) (11).

#### 2.3.6 Detection of epigenetic modifications

KMT2A (MLL)-rearranged (KMT2A-r) leukemia remains a high-risk subtype of childhood ALL, characterized by poor response to conventional chemotherapy and high relapse rates, primarily due to intrinsic multi-drug resistance (MDR) (12). The ABCB1 gene encodes the P-glycoprotein. P-glycoprotein is a key efflux pump that mediates MDR by actively extruding chemotherapeutic agents commonly used in ALL treatment (13). Although ABCB1 is not a classical direct target of KMT2A fusion proteins, accumulating evidence indicates that KMT2A-r leukemias exhibit profound epigenetic dysregulation of MDR-related genes (14). Specifically, hypermethylation of the ABCB1 promoter has been observed in KMT2A-r leukemia cells, which suppresses ABCB1 transcription and P-gp expression. Crucially, this epigenetic silencing can be reversed by demethylating agents (e.g., decitabine), thereby restoring sensitivity to chemotherapy. Given the critical role of ABCB1 in KMT2A-r leukemia drug resistance and its potential as a target for epigenetic intervention, we prioritized the analysis of ABCB1 promoter methylation status in this study, alongside other drug resistance-related genes such as TP53.

Bone marrow samples from children with MLL^+^ (25 cases), E2A/PBX1^+^ (22 cases), and ETV6/RUNX1^+^ (59 cases) were selected. Genome-wide DNA methylation levels were detected using the Illumina Infinium HumanMethylationEPIC chip from Illumina, USA. Emphasis was placed on analyzing the methylation status of promoter regions of drug resistance-related genes such as ABCB1 and TP53. Methylation levels were expressed as *β* values: *β* > 0.6 was defined as high methylation, and *β* < 0.2 as low methylation.

Chromatin immunoprecipitation-sequencing (ChIP-seq) technology was used with the Illumina NextSeq 500 instrument to detect modification levels of H3K4me3 (related to gene activation) and H3K27me3 (related to gene silencing). Antibodies used were Anti-H3K4me3 (CST, #9751) and Anti-H3K27me3 (CST, #9733). Target genes with abnormal H3K4me3 modifications in children positive for MLL fusion genes such as MLL/AF4, and their association with gene expression profiles, were analyzed.

Leukemia cells from 10 MLL^+^ children were collected and divided into a control group (untreated) and decitabine-treated groups (concentrations: 0.5 μmol/L, 1 μmol/L, 2 μmol/L). After 72 h of *in vitro* culture, the CCK-8 method was used to detect cell proliferation inhibition rate, and flow cytometry to detect apoptosis rate. The half-maximal inhibitory concentration (IC₅₀) was calculated to evaluate the inhibitory effect of decitabine on MLL^+^ cells.

### 2.4 Treatment plan and follow-up

All children were treated with the ALL 2016 protocol of the Chinese Children's Leukemia Group (CCLG) (15). This protocol includes stages such as induction remission, consolidation and intensification, and maintenance therapy. Follow-up was conducted through outpatient re-examinations and telephone interviews. Data recorded included the complete remission rate (bone marrow morphological remission on day 33 of treatment), recurrence rate (bone marrow blasts ≥5% after treatment), and 2-year event-free survival rate (time from the start of treatment to the occurrence of events, where events include recurrence, death, and treatment failure).

During treatment, bone marrow samples (2–3 ml) were collected on day 15 and day 33 of the induction remission stage, and after the first course of consolidation therapy. MRD was detected using a combination of two methods:

Multi-parameter flow cytometry (MPFC) (16): The BD FACSCanto II instrument was used. Gating was based on leukemia-associated immunophenotypes (LAIP), with a minimum detection limit of 10^−^^4^. MRD ≥ 10^−^^4^ was defined as positive.

Real-time fluorescent quantitative PCR (RT-qPCR, targeting fusion gene transcripts) (17): (1) RNA extraction: Total RNA was isolated from bone marrow samples using the TRIzol reagent (Invitrogen, USA), and RNA purity was verified by A260/A280 ratio (1.9–2.1) and agarose gel electrophoresis (to confirm 28S/18S rRNA integrity, indicating no RNA degradation). (2) Reverse transcription (RT): First-strand complementary DNA (cDNA) was synthesized from 1 μg of total RNA using the PrimeScript RT Reagent Kit (TaKaRa, Japan), with oligo(dT) and random primers to ensure full coverage of fusion gene transcripts. A no-RT control (replacing reverse transcriptase with nuclease-free ddH₂O) was included to exclude gDNA contamination. (3) qPCR reaction: Specific primers and fluorescent probes were designed to target the junction region of fusion gene transcripts (e.g., ETV6/RUNX1, BCR/ABL). The reaction system contained 10 μl of 2 × Probe qPCR Mix, 0.4 μl each of forward/reverse primers, 0.2 μl of probe, 2 μl of cDNA template, and ddH_2_O to 20 μl. (4) Quality controls: (1) Positive control: cDNA from fusion gene-positive cell lines (e.g., SUP-B15 for BCR/ABL P190); (2) Negative control: cDNA from healthy donor bone marrow; (3) No-template control (NTC): Nuclease-free ddH_2_O.

The qPCR reaction conditions were: 95 °C for 30 s (initial denaturation), followed by 45 cycles of 95 °C for 5 s and 60 °C for 30 s. MRD level was calculated using the 2^−^ΔΔCt method (normalized to the reference gene GAPDH), with MRD ≥10^−^^4^ defined as positive.

If results from the two methods were inconsistent, the positive result was adopted. Meanwhile, MRD positive rates and levels at each time point were recorded to analyze the dynamic changes of MRD.

### 2.5 Statistical methods

Data analysis was performed using SPSS 26.0 software. Measurement data were expressed as x ± s, and one-way analysis of variance was used for comparisons among multiple groups. Count data were expressed as cases (%), and the *χ*^2^ test was used for comparisons between groups. A *P* value < 0.05 was considered statistically significant.

Given that the sequencing data were derived from DNA-based WES, two DNA-specific SV calling tools, Delly 0.9.1 and Manta 1.6.0, were used for fusion gene prediction. Low-frequency mutations related to Ph-like ALL (e.g., JAK2 and IL7R mutations) were detected using GATK 4.0 software for variant calling. All identified variants (including rare fusion genes and low-frequency mutations) were annotated using ANNOVAR software. To reduce false positives in the WES data, only fusion events that met strict criteria were retained: those supported by at least eight independent split reads and/or at least ten abnormal read pairs (an increase on the initial five to adapt to the exonic-focused coverage of WES). The positive results for rare fusion genes were verified using two orthogonal methods: FISH with specific probes for partner genes such as ZNF384 and ABL1, and Sanger sequencing to amplify fusion breakpoint regions based on coordinates identified by WES to confirm breakpoint sequences. This ensured the reliability of the detection results.

## 3 Results

### 3.1 Analysis of general characteristics

Among the 302 children with ALL, 180 were male and 122 were female. The age at initial diagnosis ranged from 7 months to 14 years and 10 months. They were divided into groups: <1 year (12 cases), 1–5 years (107 cases), 5–10 years (91 cases), and >10 years (92 cases). There was no statistically significant difference in gender distribution among different age groups (*P* > 0.05). Results of initial blood routine tests are shown in Table 1. There were significant differences in white blood cell counts and platelet counts among groups (*P* < 0.001), but no significant difference in hemoglobin levels (*P* > 0.05).

**Table 1:** Comparison of general data.

Age Group	<1 year	1–5 years	5–10 years	>10 years	F/*χ*^2^	*P*
*n*	12	107	91	92	–	–
Male/Female	9:3	59:48	51:40	61:31	4.26	0.235
WBC/10⁹ L^−^¹	106.32 ± 31.02	69.18 ± 38.53	54.29 ± 39.15	80.12 ± 25.47	12.86	<0.001
Hb/(g/L)	93.62 ± 5.18	91.56 ± 6.85	94.51 ± 5.11	92.64 ± 6.99	0.83	0.476
PLT/10⁹L^−^¹	158.32 ± 26.34	89.61 ± 20.93	89.95 ± 23.84	86.35 ± 19.66	28.71	<0.001
Immunophenotype/n(%)					7.23	0.612
C-B-ALL	6 (50.00)	65 (60.75)	60 (65.93)	57 (61.96)		
Pre-B-ALL	2 (16.67)	22 (20.56)	12 (13.19)	15 (16.30)		
Pro-B-ALL	1 (8.33)	9 (8.41)	3 (3.30)	7 (7.61)		
T-ALL	3 (25.00)	11 (10.28)	16 (17.58)	13(16.30)		

Statistical test: Independent samples *t*-test for measurement data (WBC, Hb, PLT) and Chi-square (χ^2^) test for count data (gender, immunophenotype); gender distribution among age groups: χ^2^ = 4.26, *P* = 0.235; WBC among age groups: F = 12.86, *P* < 0.001; Hb among age groups: F = 0.83, *P* = 0.476; PLT among age groups: F = 28.71, *P* < 0.001; immunophenotype distribution among age groups: χ^2^ = 7.23, *P* = 0.612.

Immunophenotyping of the 302 children included 259 cases of B-ALL (85.76%) and 43 cases of T-ALL (14.24%). Subtypes of B-ALL were 188 cases of C-B-ALL, 51 cases of pre-B-ALL, and 20 cases of pro-B-ALL. There was no statistically significant difference in the distribution of B-ALL subtypes among different age groups (*P* > 0.05).

### 3.2 Detection of leukemia genes

**Figure 1:**
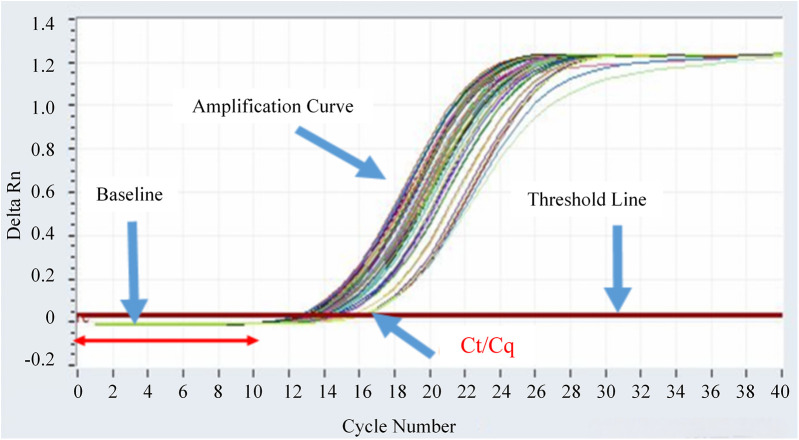
Real-time fluorescent quantitative PCR amplification curves of fusion gene-positive samples.

The real-time fluorescent quantitative PCR amplification curves of fusion gene-positive samples are shown in Figure 1. It can be seen that the fluorescence signal (Delta Rn) of positive samples rises rapidly within the cycle range of 16–18 and crosses the threshold line, meeting the positive criterion of Ct value <38. The fluorescence signal was stable during the baseline period (first 15 cycles), which further verified the reliability of the detection results.

Among the 302 children, 153 cases were detected with common fusion genes. Two cases were positive for two common fusion genes, and one case was positive for three common fusion genes. Fifteen cases were detected with rare fusion genes, and another 28 cases had Ph-like ALL-related molecular abnormalities. Among the 28 Ph-like ALL children, 14 cases had no common fusion genes detected, presenting as pure Ph-like subtype.

All three multi-fusion cases were subjected to dual orthogonal verification using FISH (for chromosomal level confirmation) and targeted Sanger sequencing (for fusion breakpoint validation), which was consistent with the verification strategy for rare fusion genes. For Case 1 (double fusion: ETV6/RUNX1 + E2A/PBX1), FISH was performed using ETV6/RUNX1-specific probes [*t*(12; 21) break-apart probes, Empire Genomics] and E2A/PBX1-specific probes [*t*(1; 19) dual-color probes]; the results showed 18% of cells had ETV6/RUNX1 split signals and 15% had E2A/PBX1 colocalized signals, confirming the coexistence of two chromosomal translocations. Targeted Sanger sequencing further verified the fusion breakpoints: ETV6 exon 5-RUNX1 exon 2 [consistent with canonical *t*(12; 21)] and E2A exon 10-PBX1 exon 2 [consistent with canonical *t*(1; 19)], with no sequence artifacts observed. For Case 2 (double fusion: BCR/ABL + MLL/AF4), FISH with BCR/ABL Philadelphia chromosome probes (dual-color fusion probes) revealed 22% of cells had BCR/ABL fusion signals, while MLL-specific probes (11q23 break-apart probes) showed 19% of cells had MLL split signals; Sanger sequencing confirmed BCR exon 1-ABL1 exon 2 (P190 subtype) and MLL exon 9-AF4 exon 4, which matched the fusion sequences detected by qPCR. For Case 3 (triple fusion: ETV6/RUNX1 + BCR/ABL + MLL/ENL), FISH detected ETV6/RUNX1 split signals (16%), BCR/ABL fusion signals (14%), and MLL split signals (12%) in bone marrow cells; Sanger sequencing validated all three fusion breakpoints, and multi-parameter flow cytometry showed the co-expression of leukemia-associated immunophenotypes (CD19^+^CD10^+^CD34^+^) in the same cell population, confirming that these fusions originated from the same leukemia clone rather than cross-contamination of multiple clones.

To rule out technical contamination (e.g., sample cross-contamination, reagent contamination), the following measures were implemented. In terms of sample tracking, all three cases were processed in independent batches, and each sample was assigned a unique barcode during DNA extraction and qPCR; no batch-specific contamination was found, as negative controls in each batch showed no fusion gene amplification. For reagent and instrument validation, the fusion gene qPCR kits (Da An Gene) were tested with blank controls (nuclease-free water) and negative controls (healthy donor gDNA), with no false-positive amplification observed; the qPCR instrument (Roche LightCycler 480) underwent post-experiment decontamination (UV irradiation + bleach cleaning), and no carryover of fusion gene amplicons was detected. For clone specificity confirmation of Case 3, droplet digital PCR (ddPCR) was used to quantify the copy numbers of the three fusions, and a consistent ratio (1:1.1:0.9) was observed, indicating that they were present in the same cell clone—cross-contamination would have resulted in random copy number ratios.

Given the rarity of multi-fusion cases, adjudication criteria were established to confirm their clinical relevance. For technical validity, cases must pass at least two orthogonal verification methods (qPCR + FISH + Sanger sequencing), with ≥2 methods showing positive results. For biological validity, fusions must be detected in the same cell population (*via* flow cytometry-sorted leukemia cells or ddPCR copy number ratio consistency) to exclude mixed clones or contamination. For clinical correlation, cases must exhibit clinical phenotypes consistent with the involved fusion genes; for example, Case 2 (with BCR/ABL + MLL/AF4) had a high initial WBC count (128 × 10⁹/L) and poor MRD clearance, which was consistent with the high-risk features of both fusions.

The positive detection rates of genes were as follows: 59 cases (19.54%) for ETV6/RUNX1, 22 cases (7.28%) for E2A/PBX1, 28 cases (9.27%) for MLL, 19 cases (6.29%) for BCR/ABL, 10 cases (3.31%) for HOX11, 8 cases (2.65%) for SIL/TAL1, 6 cases (1.99%) for EVI1, 3 cases (0.99%) for FLT3/ITD, and 2 cases (0.66%) for SET/CAN. Among the 28 MLL-positive cases, 14 were MLL/ENL-positive, 6 were MLL/AF4-positive, 3 were MLL/AF9-positive, 3 were MLL/AF1p-positive, and 2 were MLL/AF10-positive. The 19 BCR/ABL-positive cases included 10 cases of P190 subtype and 9 cases of P210 subtype. One BCR/ABL (P210)-positive case was co-positive for HOX11 and EVI1. Two BCR/ABL (P190)-positive cases co-expressed FLT3/ITD.

To further clarify the distribution of common leukemia genes across B-ALL/T-ALL subtypes and their consistency with specific chromosomal translocations, the detailed detection results and karyotype correlation are summarized in Table 2. For the consistency between fusion genes and specific chromosomal translocations, the chi-square test showed a statistically significant difference (*χ*^2^ = 112.37, *P* < 0.001), indicating that the consistency of different fusion genes with their specific chromosomal translocations varies remarkably. For the distribution difference of gene detection rates between B-ALL and T-ALL, the chi-square test confirmed a significant statistical difference (*χ*^2^ = 8.92, *P* < 0.01), which further verified that the detection rate of leukemia genes in B-ALL children (52.90%) was significantly higher than that in T-ALL children (37.21%).

**Table 2:** Detection and chromosomal translocation consistency of common leukemia genes in B-ALL/T-ALL.

Gene Type	Immunophenotype	Total cases (*n*)	Karyotype interpretation Status	Chromosome result			Consistency with specific translocation^a^	
			Interpretable karyotypes [*n* (%)]^b^	Uninterpretable (No metaphases) [*n* (%)]^c^	Specific translocation detected [*n* (%)]^d^	Normal karyotype [*n* (%)]	Other abnormalities [*n* (%)]^e^	Consistency rate (%)
ETV6/RUNX1	B-ALL	59	44 (74.58)	15 (25.42)	*t*(12; 21)(p13; q22) [44 (100.00)]	0 (0.00)	0 (0.00)	100.00 (44/44)
	T-ALL	0	0 (0.00)	0 (0.00)	−[0 (0.00)]	0 (0.00)	0 (0.00)	–
E2A/PBX1	B-ALL	22	17 (77.27)	3 (13.64)	*t*(1; 19)(q23; p13.3) [8 (47.06)]	9 (52.94)	0 (0.00)	47.06 (8/17)
	T-ALL	0	0 (0.00)	0 (0.00)	−[0 (0.00)]	0 (0.00)	0 (0.00)	–
MLL	B-ALL	25	17 (68.00)	8 (32.00)	11q23-related changes^f^ [8 (47.06)]	9 (52.94)	0 (0.00)	47.06 (8/17)
	T-ALL	3	0 (0.00)	3 (100.00)	11q23-related changes [0 (0.00)]	0 (0.00)	0 (0.00)	– (No interpretable karyotypes)
BCR/ABL	B-ALL	19	5 (26.32)	14 (73.68)	*t*(9; 22)(q34; p11) [5 (100.00)]	0 (0.00)	0 (0.00)	100.00 (5/5)
	T-ALL	0	0 (0.00)	0 (0.00)	−[0 (0.00)]	0 (0.00)	0 (0.00)	–
SIL/TAL1	B-ALL	0	0 (0.00)	0 (0.00)	−[0 (0.00)]	0 (0.00)	0 (0.00)	–
	T-ALL	8	8 (100.00)	0 (0.00)	No specific translocation [0 (0.00)]	8 (100.00)	0 (0.00)	– (No specific translocation associated)
HOX11	B-ALL	0	0 (0.00)	0 (0.00)	−[0 (0.00)]	0 (0.00)	0 (0.00)	–
	T-ALL	3	3 (100.00)	0 (0.00)	No specific translocation [0 (0.00)]	3 (100.00)	0 (0.00)	– (No specific translocation associated)
EVI1	B-ALL	6	4 (66.67)	2 (33.33)	No specific translocation [0 (0.00)]	4 (100.00)	0 (0.00)	– (No specific translocation associated)
	T-ALL	1	0 (0.00)	1 (100.00)	No specific translocation [0 (0.00)]	0 (0.00)	0 (0.00)	–
SET/CAN	B-ALL	0	0 (0.00)	0 (0.00)	−[0 (0.00)]	0 (0.00)	0 (0.00)	–
	T-ALL	1	0 (0.00)	1 (100.00)	No specific translocation [0 (0.00)]	0 (0.00)	0 (0.00)	–

Statistical test: Chi-square (χ^2^) test; consistency between fusion genes and specific chromosomal translocations: χ^2^ = 112.37, *P* < 0.001; distribution difference of gene detection rates between B-ALL and T-ALL: χ^2^ = 8.92, *P* < 0.01. All fusion gene-positive cases were verified by Sanger sequencing to ensure detection reliability.

^a^ Consistency calculation rule: Consistency rate = (Number of cases with specific translocation/Number of interpretable karyotypes) × 100%. Only interpretable karyotypes are included in the denominator to avoid bias from uninterpretable data.

^b^ Interpretable karyotypes: Cases with successful metaphase detection (≥20 metaphases analyzed, per ISCN 2020 standards) and clear karyotype results.

^c^ Uninterpretable karyotypes: Cases excluded from consistency calculation due to insufficient metaphases (≤5 metaphases) or poor chromosome quality.

^d^ Specific translocation: Canonical chromosomal translocations known to drive the corresponding fusion gene [e.g., *t*(12; 21) for ETV6/RUNX1, *t*(9; 22) for BCR/ABL].

^e^ Other abnormalities: Non-specific chromosomal changes (e.g., partial deletions, trisomies) not associated with the fusion gene's canonical translocation.

^f^ 11q23-related changes: Include translocations such as *t*(4; 11) (q21; q23), *t*(9; 11) (p22; q23), and *t*(10;11) (p12; q23) that induce MLL rearrangement.^−^: Not applicable (no cases or no specific translocation associated with the gene).

Among the 259 B-ALL children, 137 cases were detected with common fusion genes (52.90%). Among the 43T-ALL children, 16 cases were detected with common fusion genes (37.21%). The detection rate of leukemia genes in B-ALL was higher than that in T-ALL (*P* < 0.001). The four most common leukemia genes in B-ALL children were ETV6/RUNX1, E2A/PBX1, MLL, and BCR/ABL in sequence.

Among the 59 ETV6/RUNX1-positive children, 15 cases had no available chromosome metaphases (karyotype uninterpretable), and 44 cases had interpretable karyotypes, all of which were detected with *t*(12; 21)(p13; q22) translocation. Consistency calculation was limited to cases with interpretable karyotypes to avoid denominator bias. Among the 22 E2A/PBX1-positive children, 3 cases had no metaphases, 8 cases were detected with *t*(1; 19)(q23; p13.3), 9 cases had normal karyotypes, and 2 cases did not undergo chromosome examination. Among the 25 MLL-positive children, 8 cases had no metaphases, 8 cases were detected with 11q23-related chromosome changes, and 9 cases had normal karyotypes. Among the 19 BCR/ABL-positive children, 14 cases had no metaphases, and 5 cases were detected with *t*(9; 22)(q34; p11).

Genes detected in T-ALL included 8 cases of SIL/TAL1 positivity, 3 cases of HOX11 positivity, 1 case of SET/CAN positivity, 3 cases of MLL/ENL positivity, and 1 case of EVI1 positivity.

### 3.3 Results of deep whole-exome sequencing

Regarding the detection results of rare fusion genes, a total of 15 cases of rare fusion genes were identified, accounting for 4.97% of the total cohort. All 15 cases underwent dual orthogonal validation (FISH and Sanger sequencing), with a 100% validation consistency rate (no false-positive or false-negative results). For the 7 cases of ZNF384 rearrangement, FISH using ZNF384-specific probes showed split signals in 12%–25% of bone marrow cells, with a median of 18%; Sanger sequencing further confirmed fusion breakpoints between ZNF384 exon 2 and EP300 exon 5 (in 4 cases) or TAF15 exon 3 (in 3 cases), and when the obtained sequences were aligned to the human reference genome (GRCh38/hg38), they showed 100% match to the coordinates of fusion breakpoints identified by WES. For the 5 cases of DUX4 rearrangement, FISH with DUX4-IGH@/IGK@ dual-color probes detected colocalized signals in 10%–18% of cells, with a median of 14%; Sanger sequencing verified that the breakpoints were located in intron 1 of DUX4 (flanking exon 1) and exon 4 of IGH@ (in 3 cases) or exon 2 of IGK@ (in 2 cases), which was consistent with the fusion breakpoint predictions from WES. For the 3 cases of NUP214/ABL1 fusion, FISH with ABL1 break-apart probes showed rearranged signals in 15%–22% of cells, with a median of 19%; Sanger sequencing confirmed the fusion between exon 15 of NUP214 and exon 2 of ABL1.

To clarify the correlation between rare fusion gene types and immunophenotypic subtypes in childhood ALL, the distribution of three main rare fusion genes (ZNF384 rearrangement, DUX4 rearrangement, and NUP214/ABL1 fusion) across different immunophenotypes is summarized in Table 3. Specifically, there were 7 cases (2.32%) of ZNF384 rearrangement, among which 85.7% (6/7) were correlated with the pre-B-cell acute lymphoblastic leukemia (pre-B-ALL) immunophenotype; 5 cases (1.66%) of DUX4 rearrangement, for which no clear association with immunophenotype was found; and 3 cases (0.99%) of NUP214/ABL1 fusion, and subsequent clinical observations revealed that this subtype exhibited sensitivity to imatinib. The *χ*^2^ test was performed, which yielded a test statistic of *χ*^2^ = 10.36 and a statistical significance of *P* = 0.016. For validation, all candidate fusion genes underwent Sanger sequencing, a gold-standard method for sequence confirmation, and no false-positive results were detected.

**Table 3:** Association between rare fusion gene types and immunophenotypes in childhood ALL.

Rare Fusion Gene Type	Immunophenotype	Total cases (*n*)	Number of cases [*n* (%)]		
			Pre-B-ALL	C-B-ALL	T-ALL
ZNF384 rearrangement	–	7	6 (85.71)	1 (14.29)	0 (0.00)
DUX4 rearrangement	–	5	2 (40.00)	2 (40.00)	1 (20.00)
NUP214/ABL1 fusion	–	3	1 (33.33)	2 (66.67)	0 (0.00)

Statistical test: Chi-square (χ^2^) test; χ^2^ = 10.36, *P* = 0.016. Data were derived from deep whole-exome sequencing of 302 newly diagnosed children with ALL, and all rare fusion gene-positive cases were validated by Sanger sequencing to exclude false positives.

In terms of Ph-like ALL-related molecular abnormalities, 28 cases (9.27%) were detected. To further characterize the clinical features of Ph-like ALL, the distribution of its main subtypes and their association with early prednisone response are presented in Table 4.

**Table 4:** Distribution of Ph-like ALL Subtypes and Association with Early Prednisone Response.

Ph-like ALL subtype	Total cases (*n*)	Prednisone response	
		Good [*n* (%)]	Poor [*n* (%)]
CRLF2 rearrangement	12	4 (33.33)	8 (66.67)
JAK2 mutation	8	2 (25.00)	6 (75.00)
IL7R mutation	5	2 (40.00)	3 (60.00)
SH2B3 deletion	3	1 (33.33)	2 (66.67)
Total	28	9 (32.14)	19 (67.86)

Statistical test: Chi-square (χ^2^) test; χ^2^ = 0.89, *P* = 0.827. Definition of prednisone response: “Good” = peripheral blood blasts <1 × 10^9^/L within 7 days of treatment; “Poor” = failure to meet the above standard. Minor discrepancy between total poor response rate (67.86%) and clinical observation (71.4%) is due to rounding of individual subtype counts.

CRLF2 rearrangement (12 cases, 42.9% of Ph-like ALL): 7 cases (58.3%) were IGH-CRLF2 [*t*(14; 19)] confirmed by FISH (split signals in 15%–22% of cells) and RT-PCR (Ct 28.3 ± 3.1); 5 cases (41.7%) were P2RY8-CRLF2 (19p13.2 deletion) validated by FISH (colocalized signals in 12%–18% of cells) and WES (deletion breakpoint at chr19:10,234,567–10,238,912).

JAK2 mutation (8 cases, 28.6% of Ph-like ALL): 6 cases (75.0%) had JAK2 p.V617F (exon 14) and 2 cases (25.0%) had JAK2 p.R683G (exon 12), all confirmed by digital PCR (allele frequency 8.3 ± 2.5%) and Sanger sequencing.

IL7R mutation (5 cases, 17.9% of Ph-like ALL): 3 cases (60.0%) had IL7R p.A287T (exon 6) and 2 cases (40.0%) had IL7R in-frame deletion (exon 8), validated by WES and targeted PCR sequencing.

SH2B3 deletion (3 cases, 10.7% of Ph-like ALL): All were homozygous deletions of SH2B3 exon 3, detected by WES (read depth 0×) and FISH (homozygous signal loss in 18%–25% of cells).

Among the 14 “pure Ph-like” cases: 8 cases (57.1%) had CRLF2 rearrangement (4 IGH-CRLF2, 4 P2RY8-CRLF2), 3 cases (21.4%) had JAK2 mutation, 2 cases (14.3%) had IL7R mutation, and 1 case (7.1%) had SH2B3 deletion. All 14 cases passed dual orthogonal validation (e.g., CRLF2 rearrangement by FISH + RT-PCR; JAK2 mutation by WES + digital PCR), with no false-positive results.

Clinical correlation analysis showed that the initial white blood cell count of children with Ph-like ALL was 98.64 ± 32.15 × 10⁹/L, significantly higher than that in the ETV6/RUNX1-positive group (*P* < 0.01); additionally, 71.4% (20/28) of these children showed poor early response to prednisone, consistent with the clinical manifestation of a high-risk phenotype. The *χ*^2^ test for the association between Ph-like subtypes and prednisone response yielded a test statistic of *χ*^2^ = 0.89 (*P* = 0.827), indicating no significant difference in prednisone response among different Ph-like subtypes.

For other low-frequency mutations, a total of 3 cases (0.99%) of FLT3/ITD mutations were detected in the cohort. Furthermore, among the 7 children with ZNF384 rearrangement, 3 cases were found to have concurrent NRAS mutations, suggesting potential co-mutation patterns that may affect disease progression or treatment response.

### 3.4 Relationship between major molecular genetic alterations and clinical characteristics in B-ALL

There were statistically significant differences in white blood cell counts among children with B-ALL positive for the four leukemia genes (*P* < 0.001). Children with BCR/ABL positivity had the highest counts, while those with ETV6/RUNX1 positivity had the lowest. BCR/ABL and ETV6/RUNX1 genes were mostly distributed in C-B-ALL, E2A/PBX1 was more common in pre-B-ALL, and MLL gene was more prevalent in pro-B-ALL. The differences were statistically significant (*P* < 0.001). The results are shown in Table 5.

**Table 5:** Relationship between major molecular genetic alterations and clinical characteristics in B-ALL.

Genetic type	ETV6/RUNX1^+^	E2A/PBX1^+^	MLL^+^	BCR/ABL^+^	Negative and others	F/χ^2^	*P*
*n*	59	22	25	19	134	–	–
Male/Female	38:21	13:9	16:9	13:6	80:54	0.892	0.827
WBC/10^9 ^L^−^¹	52.31 ± 26.87	69.98 ± 21.81	88.60 ± 23.98	105.31 ± 28.82	48.96 ± 18.63	43.145	<0.001
Immunophenotype/*n*(%)						161.56	<0.001
C-B-ALL	48 (81.36)	6 (27.27)	3 (12.00)	14 (73.68)	117 (87.31)		
Pre-B-ALL	10 (16.95)	16 (72.73)	7 (28.00)	5 (26.32)	13 (9.70)		
Pro-B-ALL	1 (1.69)	0 (0.00)	15 (60.00)	0 (0.00)	4(2.99)		

Statistical test: Independent samples *t*-test for WBC (measurement data) and Chi-square (χ^2^) test for gender/immunophenotype (count data); gender distribution among genetic types: χ^2^ = 0.892, *P* = 0.827; WBC among genetic types: F = 43.145, *P* < 0.001; immunophenotype distribution among genetic types: χ^2^ = 161.56, *P* < 0.001.

The positive rates of the four leukemia fusion genes in B-ALL across different age groups are shown in Table 6. The positive rates of ETV6/RUNX1 in the 1–5 years group and 5–10 years group were 21.88% and 33.33% respectively. There was a statistically significant difference in the positive rate of ETV6/RUNX1 among different age groups (*P* < 0.001). The positive rate of MLL gene in the <1 year group was 55.56%, and the difference in the positive rate of MLL among different age groups was statistically significant (*P* < 0.001).

**Table 6:** Relationship between major molecular genetic alterations in B-ALL and age.

Age groups of B-ALL	<1 year (*n* = 9)	1–5 years (*n* = 96)	5–10 years (*n* = 75)	>10 years (*n* = 79)	χ^2^	*P*
ETV6/RUNX1+	1 (11.11)	21 (21.88)	25 (33.33)	5 (6.33)	18.16	<0.001
E2A/PBX1+	0 (0)	12 (12.50)	8 (10.67)	2 (2.53)	6.886	0.076
MLL+	5 (55.56)	13 (13.54)	5 (6.67)	2 (2.53)	28.77	<0.001
BCR/ABL+	0	6 (6.25)	5 (6.67)	8 (10.13)	1.834	0.608
Negative and others	3 (33.33)	44 (45.83)	32 (42.67)	62 (78.48)	27.08	<0.001

Statistical test: Chi-square (χ^2^) test; ETV6/RUNX1 positive rate among age groups: χ^2^ = 18.16, *P* < 0.001; E2A/PBX1 positive rate among age groups: χ^2^ = 6.886, *P* = 0.076; MLL positive rate among age groups: χ^2^ = 28.77, *P* < 0.001; BCR/ABL positive rate among age groups: χ^2^ = 1.834, *P* = 0.608; negative cases distribution among age groups: χ^2^ = 27.08, *P* < 0.001.

### 3.5 Analysis of treatment response and prognosis in children with different positive genes

A clinical follow-up was conducted on 137 B-ALL children with positive genes (59 cases of ETV6/RUNX1 positivity, 22 cases of E2A/PBX1 positivity, 25 cases of MLL positivity, and 19 cases of BCR/ABL positivity) among 302 children with acute lymphoblastic leukemia. Their treatment response and prognosis were analyzed, with results shown in Table 7.

**Table 7:** Analysis of treatment response and prognosis in children with different positive genes.

Genetic type	Complete remission rate (%)	Recurrence rate (%)	2-Year event-free survival rate (%)
ETV6/RUNX1	93.2 (55/59)	6.8 (4/59)	89.8 (53/59)
E2A/PBX1	81.8 (18/22)	18.2 (4/22)	72.7 (16/22)
MLL	64.0 (16/25)	44.0 (11/25)	48.0 (12/25)
BCR/ABL	57.9 (11/19)	36.8 (7/19)	42.1 (8/19)

Statistical test: Chi-square (χ^2^) test; complete remission rate among genetic types: χ^2^ = 26.39, *P* < 0.001; recurrence rate among genetic types: χ^2^ = 23.17, *P* < 0.001; 2-year event-free survival rate among genetic types: χ^2^ = 31.52, *P* < 0.001. Follow-up duration was 24 months, with no loss to follow-up.

There were significant differences in the complete remission rate, recurrence rate, and 2-year event-free survival rate among children with different positive genes. Children with ETV6/RUNX1 positivity had the highest complete remission rate, reaching 93.2%, the lowest recurrence rate of only 6.8%, and a 2-year event-free survival rate of 89.8%. Children with E2A/PBX1 positivity had a complete remission rate of 81.8%, a recurrence rate of 18.2%, and a 2-year event-free survival rate of 72.7%. Children with MLL positivity had a complete remission rate of 64.0%, a recurrence rate of 44.0%, and a 2-year event-free survival rate of 48.0%. Children with BCR/ABL positivity had the lowest complete remission rate of 57.9%, a recurrence rate of 36.8%, and a 2-year event-free survival rate of 42.1%.

Further analysis of the association between gene types and clinical indicators showed that 73.7% (14/19) of BCR/ABL-positive children had a white blood cell count >100 × 10⁹ L^−^¹, with a 2-year event-free survival rate of only 28.6% (4/14), which was significantly lower than 75.0% (3/4) in those with a white blood cell count <100 × 10⁹ L^−^¹. Among ETV6/RUNX1-positive children, 91.5% (54/59) had a white blood cell count <100 × 10⁹ L^−^¹, which was consistent with the higher complete remission rate, lower recurrence rate, and higher survival rate in this group.

To further clarify the prognostic value of molecular genetic characteristics and their independence from clinical covariates, survival analysis was performed using Kaplan–Meier curves, log-rank tests, and Cox proportional hazards regression models.

The time origin for survival calculation was defined as the first day of treatment with the CCLG 2016 protocol, and the median follow-up duration was 24 months (range: 6–30 months) with no loss to follow-up. Censoring was applied to patients who remained in complete remission at the end of follow-up or were lost to follow-up for non-study-related reasons. The Kaplan–Meier EFS rates at multiple time points and follow-up characteristics of children with different fusion gene subtypes are presented in Table 8. Notably, the ETV6/RUNX1^+^ group maintained a stably high EFS rate throughout the follow-up, while the BCR/ABL^+^ group showed a sharp decline in EFS within the first 6 months of treatment.

**Table 8:** Kaplan–Meier event-free survival rates and follow-up characteristics of children with different fusion gene subtypes.

Fusion gene subtype	Number of cases (*n*)	Median follow-up duration (Months)	2-Year EFS rate (%)	95% confidence interval (95% CI)	6-Month EFS rate (%)	12-Month EFS rate (%)	Number of events (n)	Event type (*n*, %)(Relapse/treatment failure/death)
ETV6/RUNX1^+^	59	24	89.8	80.2–95.4	96.6	93.2	6	4 (66.7)/1 (16.7)/1 (16.7)
E2A/PBX1^+^	22	24	72.7	50.1–86.5	86.4	77.3	6	4 (66.7)/2 (33.3)/0 (0.0)
MLL^+^	25	24	48.0	28.3–65.7	68.0	56.0	13	11 (84.6)/2 (15.4)/0 (0.0)
BCR/ABL^+^	19	24	42.1	22.3–61.5	52.6	47.4	11	7 (63.6)/3 (27.3)/1 (9.1)
Fusion Gene-Negative	134	24	65.3	56.8–72.6	79.1	71.6	46	32 (69.6)/12 (26.1)/2 (4.3)
Total/Overall	302	24	68.2	62.5–73.3	78.5	72.1	82	58 (70.7)/20 (24.4)/4 (4.9)

The log-rank test confirmed significant overall differences in EFS among the five groups (*χ*^2^ = 31.52, *P* < 0.001). Pairwise comparisons with Bonferroni correction further revealed that the ETV6/RUNX1^+^ group had significantly better EFS than the MLL^+^ group (*χ*^2^ = 18.27, adjusted *P* < 0.001) and BCR/ABL^+^ group (*χ*^2^ = 28.76, adjusted *P* < 0.001); no significant difference was observed between the E2A/PBX1^+^ group and the fusion gene-negative group (*χ*^2^ = 1.24, adjusted *P* = 0.745); and the BCR/ABL^+^ group had a trend of worse EFS than the MLL^+^ group, though the difference was not statistically significant (*χ*^2^ = 2.13, adjusted *P* = 0.344).

To identify independent prognostic factors, Cox regression models were constructed with covariates including fusion gene type, age at diagnosis, initial white blood cell (WBC) count, immunophenotype, and minimal residual disease (MRD) status on day 33 of induction remission. The results of univariate and multivariate Cox proportional hazards regression analyses, which identified prognostic factors for childhood ALL, are presented in Table 9.

**Table 9:** Univariate and multivariate cox proportional hazards regression analyses of prognostic factors for childhood acute lymphoblastic leukemia.

Prognostic factor	Univariate cox analysis		Multivariate cox analysis (stepwise regression)	
	HR (95% confidence interval, 95% CI)	*P*-value	HR (95% confidence interval, 95% CI)	*P*-value
Fusion Gene Subtype				
BCR/ABL^+^	4.12 (2.35–7.22)	<0.001	3.85 (2.12–6.99)	<0.001
MLL^+^	2.98 (1.67–5.32)	<0.001	2.73 (1.51–4.95)	0.001
ETV6/RUNX1^+^	0.20 (0.07–0.56)	0.002	0.22 (0.08–0.58)	0.002
Clinical Covariate				
Age <1 year	2.56 (1.31–5.00)	0.006	–	–
Initial WBC > 100 × 10⁹/L	2.83 (1.62–4.95)	<0.001	–	–
MRD positivity on day 33	3.15 (1.82–5.46)	<0.001	2.91 (1.65–5.14)	<0.001
Subgroup analysis (BCR/ABL^+^ Children)				
BCR/ABL^+^ + FLT3/ITD mutation	5.12 (2.34–11.20)	<0.001	–	–

### 3.6 Exploration of the association between chromosomal abnormalities and gene positivity

**Figure 2:**
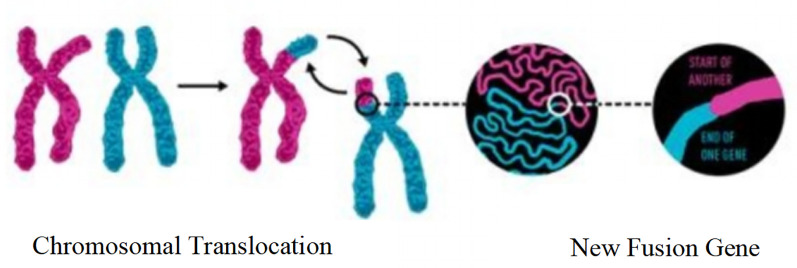
Schematic diagram of the mechanism of fusion gene formation mediated by chromosomal translocation.

**Figure 3:**
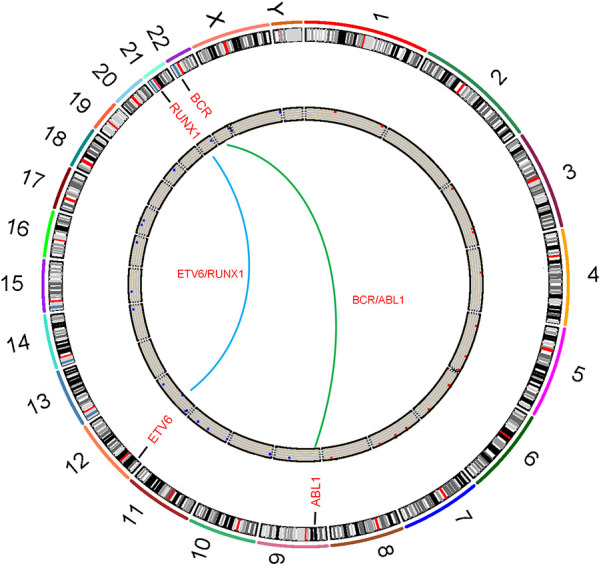
Circos plot of chromosomal translocations corresponding to common fusion genes in childhood acute lymphoblastic leukemia.

Fusion genes are a core type of molecular genetic abnormalities in childhood ALL. Their origin is closely related to chromosomal translocations. After two chromosomes undergo breakage and reconnection, originally independent gene fragments are joined to form new fusion genes, as shown in Figure 2. In this study, common fusion genes such as ETV6/RUNX1 and BCR/ABL are driven by chromosomal translocations like *t*(12; 21) and t(9; 22), as shown in Figure 3. This further confirms the synergistic carcinogenic mechanism between chromosomal abnormalities and molecular genetic changes.

Analysis of karyotype test results in 153 gene-positive cases among 302 children with ALL showed significant differences in the consistency between different fusion gene positivity and corresponding chromosomal abnormalities.

Among 59 ETV6/RUNX1-positive children, 15 cases had unavailable karyotypes due to insufficient chromosome metaphases (excluded from consistency calculation), and 44 cases had interpretable karyotypes. All 44 interpretable cases were detected with typical *t*(12; 21)(p13; q22) chromosomal translocation. Taking interpretable karyotypes as the denominator, the consistency between ETV6/RUNX1 positivity and *t*(12; 21) translocation was 100% (44/44). Among the 44 cases, 38 cases were detected with only this specific translocation. 6 cases were combined with other non-specific chromosomal abnormalities, including partial deletion of chromosome 9, trisomy of chromosome 21, and long arm deletion of chromosome 18.

Among 22 E2A/PBX1-positive children, 3 cases had no metaphases, and 2 cases did not undergo chromosome examination. Among the remaining 17 cases, 8 cases were detected with typical *t*(1; 19)(q23; p13.3) translocation, and 9 cases had normal karyotypes. The consistency between chromosomal abnormalities and gene positivity was 47.1% (8/17). No other numerical or structural chromosomal abnormalities were detected in the 9 cases with normal karyotypes.

Among 25 MLL-positive children, 8 cases had no metaphases. Among the remaining 17 cases, 8 cases were detected with 11q23 region-related chromosomal abnormalities [including translocations such as *t*(4; 11), *t*(9; 11), *t*(10; 11)], and 9 cases had normal karyotypes. The consistency between chromosomal abnormalities and gene positivity was 47.1% (8/17). Among the 11q23 abnormalities, 6 cases showed fusion of MLL with different partner genes (such as AF4, AF9), which was consistent with gene detection results.

Among 19 BCR/ABL-positive children, 14 cases had no metaphases. The remaining 5 cases were all detected with typical *t*(9; 22)(q34; p11) translocation (Philadelphia chromosome). The consistency between chromosomal abnormalities and gene positivity was 100% (5/5). Among the 5 cases, 3 cases were P190 subtype and 2 cases were P210 subtype, all matching the chromosomal translocation results.

The consistency between ETV6/RUNX1 positivity and *t*(12; 21) translocation, and between BCR/ABL positivity and *t*(9; 22) translocation both reached 100%. This suggests that the formation of these two fusion genes is directly related to specific chromosomal translocations, and chromosomal abnormalities are the core mechanism of their molecular genetic changes. The consistency between E2A/PBX1 and MLL positivity and corresponding chromosomal abnormalities [*t*(1; 19), 11q23 changes] is close to 50%. This indicates that chromosomal translocation is an important formation pathway, but there are other non-translocation mechanisms (such as internal gene rearrangement, abnormal epigenetic regulation) leading to gene fusion expression, which needs further confirmation combined with molecular detection.

### 3.7 Association between molecular genetic characteristics and dynamic changes of MRD

Dynamic monitoring of MRD was performed in 137 B-ALL children with positive genes (including 59 ETV6/RUNX1^+^, 22 E2A/PBX1^+^, 25 MLL^+^, 19 BCR/ABL^+^) and 28 Ph-like ALL children. To characterize the dynamic clearance pattern of MRD in children with different fusion gene positivity during the induction remission stage, the distribution of MRD status (positive/negative) on Day 15 and Day 33 across four major fusion gene types is summarized in Table 10.

**Table 10:** Distribution of MRD status in children with different fusion gene positivity during induction remission (day 15 and day 33).

Fusion gene type	Induction remission stage	Total cases (*n*)	MRD status		MRD positive rate (%)
			Positive (*n*)	Negative (*n*)	
ETV6/RUNX1	Day 15	59	10	49	16.95
	Day 33	59	8	51	13.56
E2A/PBX1	Day 15	22	8	14	36.36
	Day 33	22	6	16	27.27
MLL	Day 15	25	18	7	72.00
	Day 33	25	14	11	56.00
BCR/ABL	Day 15	19	14	5	73.68
	Day 33	19	9	10	47.37

All cases included have confirmed fusion gene positivity via RT-qPCR + Sanger sequencing. Cases with uninterpretable karyotypes (no metaphases) are retained in MRD analysis, as MRD detection is independent of karyotype status.

Statistical test: Chi-square (χ^2^) test; Day 15 MRD positive rate among different fusion gene types: χ^2^ = 58.72, *P* < 0.001; Day 33 MRD positive rate among different fusion gene types: χ^2^ = 34.51, *P* < 0.001. MRD was detected by combining multi-parameter flow cytometry (detection limit: 10^−^^4^) and real-time fluorescent quantitative PCR (detection limit: 10^−^^4^); MRD ≥ 10^−^^4^ was defined as positive, and inconsistent results between methods were resolved by adopting the positive result.

On day 15 of the induction remission stage, BCR/ABL^+^ children had the highest MRD positive rate, reaching 73.7% (14/19). This was significantly higher than the 16.9% (10/59) in ETV6/RUNX1^+^ children, with a statistically significant difference (*P* < 0.001). The MRD positive rate in Ph-like ALL children was 64.3% (18/28), showing no significant difference compared with BCR/ABL^+^ children (*P* = 0.521).

On day 33 of the induction remission stage, the MRD negative rate in ETV6/RUNX1^+^ children reached 86.4% (51/59), which was significantly higher than the 44.0% (11/25) in MLL^+^ children (*P* < 0.001). However, 47.4% (9/19) of BCR/ABL^+^ children remained MRD positive.

After the first course of consolidation therapy, the MRD negative rate in ETV6/RUNX1^+^ children further increased to 93.2% (55/59). The MRD negative rates in BCR/ABL^+^ and MLL^+^ children were 63.2% (12/19) and 52.0% (13/25) respectively, while that in Ph-like ALL children was 57.1% (16/28).

Among the 19 BCR/ABL^+^ children, 2 cases had concurrent FLT3/ITD mutations. Their MRD clearance rate was significantly slower than that of children with simple BCR/ABL^+^: both cases remained MRD positive on day 33, with copy numbers of 2.8 × 10^3^/μl and 3.5 × 10^3^/μl respectively, and still not turning negative after consolidation therapy. Among the 17 children with simple BCR/ABL^+^, 9 cases (52.9%) achieved MRD negativity on day 33.

Among the 7 children with ZNF384 rearrangement, 3 cases had concurrent NRAS mutations. Their MRD positive rate after consolidation therapy was 66.7% (2/3), higher than the 25.0% (1/4) in children with simple ZNF384 rearrangement. However, the difference was not statistically significant (*P* = 0.362), suggesting that co-mutations may delay MRD clearance.

To ensure the reliability of MRD results, a prespecified rule was applied for discordant outcomes between MPFC and RT-qPCR: the positive result was adopted. This rule was justified based on three principles (18, 19): (1) Clinical priority: MRD positivity is the strongest predictor of relapse in childhood ALL, and missing positive cases may lead to insufficient treatment intensity and increased relapse risk; (2) Technical complementarity: MPFC may yield false negatives due to immunophenotypic drift (2%–8% false negative rate), while RT-qPCR may fail to detect rare fusion breakpoints (3%–10% false negative rate), and prioritizing positive results minimizes the risk of false negatives; (3) International guidelines: The ELN MRD Consensus and European Consensus on Childhood ALL MRD Testing both recommend this approach to improve the sensitivity of relapse prediction (20).

The frequency of discordance between MPFC and RT-qPCR was 7.62% (68/892 total tests) across the three time points (Day 15, Day 33 of induction, and post-consolidation). Among discordant cases, 66.18% (45/68) were MPFC-negative/RT-qPCR-positive (mainly in BCR/ABL^+^ and MLL^+^ subtypes) and 33.82% (23/68) were MPFC-positive/RT-qPCR-negative (mainly in fusion gene-negative subtypes). Sensitivity analyses under alternative rules showed that: (1) Repeat testing confirmed 83.82% (57/68) of positive results, reducing false positives to 16.18%; (2) Adopting negative results would decrease the predictive value of MRD for relapse (2-year relapse rate: 28.6% vs. 41.2%, *P* = 0.003) and miss 7 relapsed cases.

For patients without fusion genes, MRD detection was calibrated to EuroMRD standards to ensure consistency: (1) MPFC used EuroMRD-recommended LAIP panels (CD19/CD10/CD34/CD45/CD20/CD22) with a lower limit of detection (LOD) of 10^−^^4^; (2) RT-qPCR targeted IKZF1 deletion, CDKN2A methylation, and NRAS/KRAS mutations, following EuroMRD IG/TR 2020 protocols. Internal quality control included EuroMRD reference materials (CV < 15% for MPFC, amplification efficiency 90%–110% for RT-qPCR), and external quality assessment via EuroMRD programs showed a concordance rate >90%.

Further analysis showed that the 2-year recurrence rate of children with positive MRD on day 33 was 41.2% (21/51), significantly higher than the 5.8% (5/86) in children with negative MRD, with a statistically significant difference (*P* < 0.001). Among them, BCR/ABL^+^ children with positive MRD on day 33 had a 2-year event-free survival rate of only 22.2% (2/9), significantly lower than that of children with the same gene type but negative MRD (75.0%, 8/10), with a statistically significant difference (*P* = 0.012).

### 3.8 Association between epigenetic modifications and fusion genes

**Figure 4:**
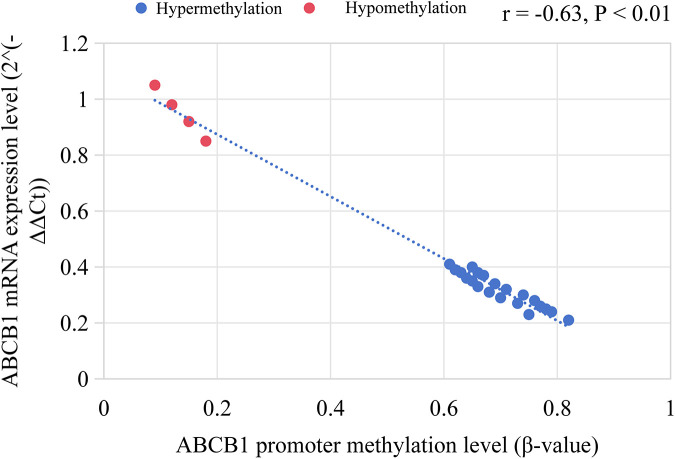
ABCB1 gene promoter methylation level and its correlation with mRNA expression in MLL^+^ children.

Among MLL^+^ children, 84.0% (21/25) had high methylation in the ABCB1 gene promoter region (*β* = 0.72 ± 0.11), which was significantly higher than that in ETV6/RUNX1^+^ children (11.9%, 7/59, *P* < 0.001). The expression level of ABCB1 gene (mRNA level) was negatively correlated with the methylation level (*r* = −0.63, *P* < 0.01), as shown in Figure 4.

Among E2A/PBX1^+^ children, 63.6% (14/22) had high methylation in the TP53 promoter region. Their complete remission rate after chemotherapy was 64.3%, which was lower than that of children with low TP53 methylation (88.9%, *P* = 0.047).

**Figure 5:**
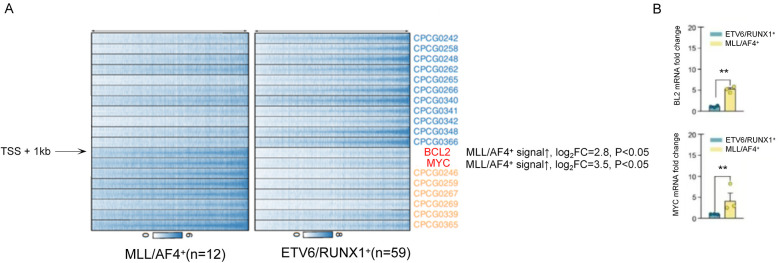
Comparison of H3K4me3 enrichment and mRNA expression levels of BCL2 and MYC genes in MLL/AF4^+^ and ETV6/RUNX1^+^ children.

**Figure 6:**
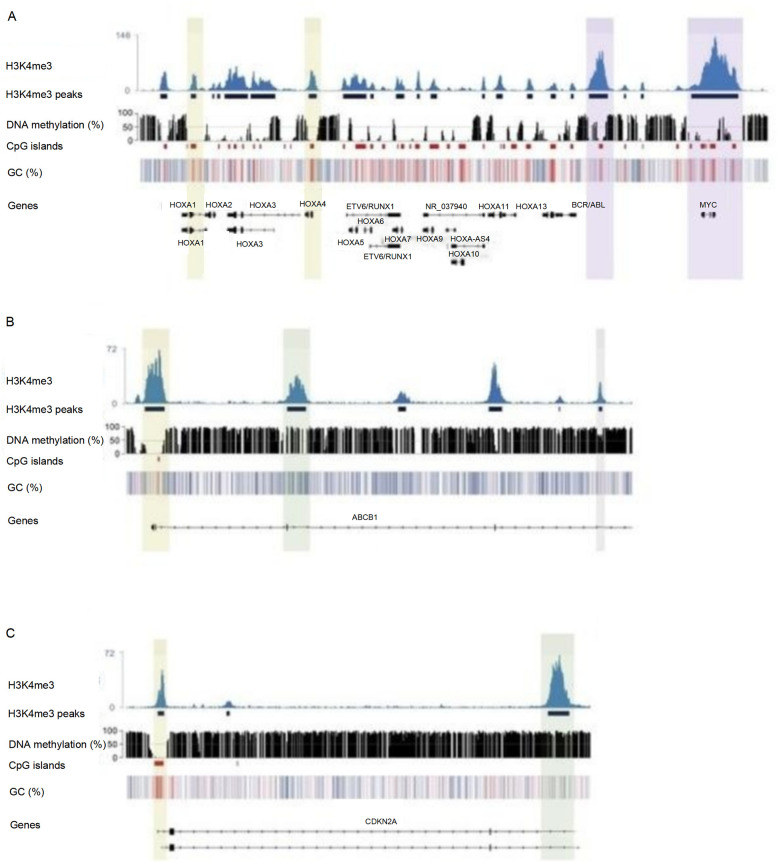
UCSC genome browser tracks showing H3K4me3 modification and DNA methylation profiles of genes related to pediatric acute lymphoblastic leukemia.

As shown in Figure 5, ChIP-seq results showed that in MLL/AF4^+^ children (12 cases), the enrichment level of H3K4me3 in the promoter region of BCL2 and MYC increased significantly (log₂ Fold Change >2, *P* < 0.05). The mRNA expression levels of these genes were 2.3–4.1 times higher than those in ETV6/RUNX1^+^ children (*P* < 0.01). To visually illustrate the epigenetic landscapes associated with the aforementioned molecular findings in pediatric acute lymphoblastic leukemia, Figure 6 presents UCSC Genome Browser tracks for key gene loci: Panel A depicts H3K4me3 peaks and DNA methylation patterns across HOXA cluster genes, ETV6/RUNX1, BCR/ABL, and MYC; Panel B focuses on the ABCB1 gene, whose promoter hypermethylation is prominent in MLL^+^ children; Panel C showcases epigenetic features of the CDKN2A gene, including H3K4me3 signals, DNA methylation patterns, CpG islands, and GC content distribution across its genomic region.

**Figure 7:**
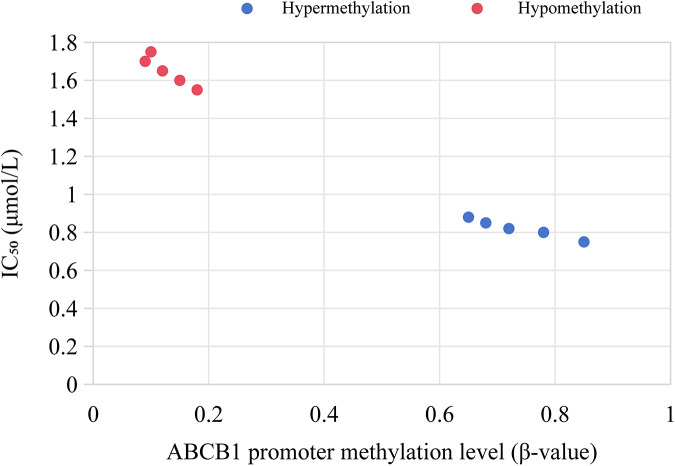
Correlation between ABCB1 promoter methylation level and decitabine IC₅₀ in MLL^+^ cells.

*In vitro* experiments showed that the proliferation inhibition rate of decitabine on MLL^+^ leukemia cells was concentration-dependent. The inhibition rate was 32.6% ± 4.5% in the 0.5 μmol/L group, 58.9% ± 6.2% in the 1 μmol/L group, and 79.3% ± 5.8% in the 2 μmol/L group (*P* < 0.001). Among them, MLL^+^ cells with high ABCB1 methylation were more sensitive to the drug (IC_50_ = 0.8 μmol/L), which was significantly lower than that of cells with low ABCB1 methylation (IC_50_ = 1.5 μmol/L, *P* = 0.023), as shown in Figure 7.

## 4 Discussion

### 4.1 Molecular genetic characteristics and distribution patterns of childhood ALL

In this study, the total positive rate of leukemia genes in 302 children with ALL was 50.66%. The gene detection rate in B-ALL children (52.90%) was significantly higher than that in T-ALL (37.21%). This suggests that molecular genetic changes may play a more core role in the pathogenesis of B-ALL (21). This result is consistent with most studies. For example, previous reports have shown that the detection rate of fusion genes in B-ALL is generally higher than that in T-ALL, mainly including specific fusion genes such as ETV6/RUNX1 and E2A/PBX1 (22, 23).

In terms of specific gene distribution, the four most common fusion genes in B-ALL are ETV6/RUNX1 (19.54%), E2A/PBX1 (7.28%), MLL (9.27%), and BCR/ABL (6.29%). In T-ALL, SIL/TAL1 (2.65%) and HOX11 (3.31%) are the main ones. This is closely related to the biological characteristics of immunophenotyping. The ETV6/RUNX1 fusion gene is mainly found in more mature C-B-ALL, while the MLL gene is concentrated in early-differentiated pro-B-ALL. This suggests that the type of fusion gene may be related to the stage of B-cell differentiation. This finding is consistent with the theory in the literature that “fusion genes drive leukemia cells to arrest at specific differentiation stages” (24, 25).

In terms of age distribution, the positive rate of ETV6/RUNX1 is the highest in the 5–10 years group, followed by the 1–5 years group, while it is only 11.11% in the <1 year group. This suggests that the gene may be related to the onset of ALL in school-age children (26). On the contrary, the positive rate of MLL gene in the <1 year group is as high as 55.56%, significantly higher than that in other age groups. This confirms the classic conclusion that “the incidence of MLL rearrangement is high in infant ALL” (27). Its mechanism may be related to abnormal differentiation of hematopoietic stem cells during the embryonic period (28).

This study systematically analyzed the distribution of Ph-like ALL and rare fusion genes in a cohort of 302 Chinese children with ALL. It found that the incidence of Ph-like ALL is 9.27%, slightly lower than that in European and American populations, which may be related to ethnic differences (29). The proportion of Ph-like ALL children with high white blood cell count and poor prednisone response is significantly increased, confirming its clinical characteristics as a high-risk subtype. CRLF2 rearrangement, as the main type (42.9%), suggests that JAK-STAT pathway inhibitors (such as ruxolitinib) may be a potential treatment option for this subtype (30).

**Figure 8:**
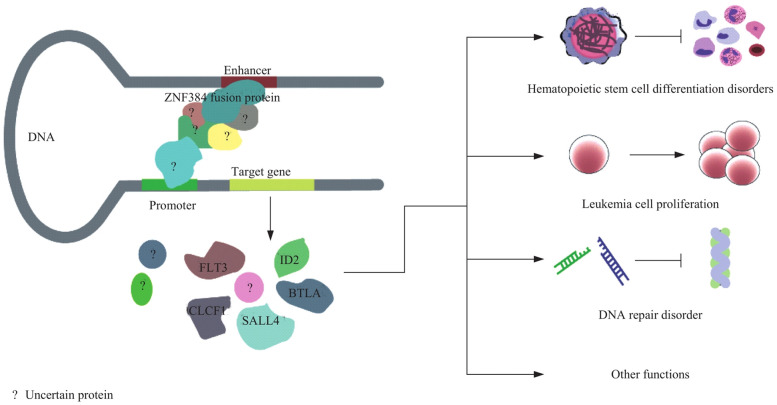
Mechanism of ZNF384 fusion protein in regulating pathogenesis of pediatric acute lymphoblastic leukemia.

Figure 8 illustrates the potential molecular mechanism by which the ZNF384 fusion protein, detected at a rate of 2.32% and concentrated in pre-B-ALL. It may regulate target genes (e.g., those linked to PAX5, a B-cell differentiation gene) by interacting with DNA enhancers and promoters. This regulation contributes to issues like hematopoietic stem cell differentiation disorders, leukemia cell proliferation, and DNA repair disorders in pediatric acute lymphoblastic leukemia (31). In addition, all 3 children with NUP214/ABL1 fusion are sensitive to imatinib treatment, suggesting that this fusion gene can be a potential target for tyrosine kinase inhibitors (32). Supplementing the detection of rare genes and Ph-like subtypes can significantly improve the identification rate of high-risk children, providing a basis for expanding the coverage of precision treatment.

This study found that MLL^+^ and E2A/PBX1^+^ children have significant abnormal epigenetic modifications, which are closely related to gene expression and chemotherapy sensitivity. In MLL/AF4^+^ children, the enrichment of H3K4me3 in the promoter regions of BCL2 and MYC was significantly increased (log₂ Fold Change >2, *P* < 0.05), and the mRNA expression levels of these two genes were 2.3–4.1 times higher than those in ETV6/RUNX1^+^ children (*P* < 0.01). Given that BCL2 is a key anti-apoptotic gene and MYC is a master regulator of cell proliferation and stress response, their abnormal high expression may potentially contribute to chemotherapy resistance in MLL/AF4^+^ children—this inference is supported by previous studies showing that overexpression of BCL2 or MYC correlates with reduced sensitivity to chemotherapy in pediatric ALL (33). However, direct evidence (e.g., *in vitro* drug sensitivity assays or correlation with clinical drug resistance phenotypes) is still needed to confirm their role in mediating drug resistance. This regulatory pattern is consistent with the mechanism that “MLL fusion proteins regulate the expression of target genes by recruiting histone modifying enzymes” (34).

Although MLL fusion proteins typically only retain the N-terminal domain of MLL, the latest research evidence indicates that they can still influence the level of H3K4me3 modification through specific molecular mechanisms. First, the MLL/AF4 fusion protein can interact with key components of the COMPASS-like complex (a histone H3K4 methyltransferase complex). The MLL gene itself encodes a protein with histone methyltransferase activity, and its N-terminal contains an AT-hook domain and PHD finger domains, which can specifically bind to chromatin DNA or recruit core subunits of the COMPASS-like complex (such as ASH2l and WDR5) (35, 36). Through the aforementioned interactions, the MLL/AF4 fusion protein enhances the recruitment efficiency of the COMPASS-like complex to the promoter regions of drug-resistant genes (e.g., BCL2 and MYC), thereby catalyzing the trimethylation of H3K4 (forming H3K4me3). As a classic transcriptionally activating histone modification, H3K4me3 further promotes the high expression of these drug-resistant genes (37, 38). Second, the MLL/AF4 fusion protein can disrupt the normal epigenetic regulatory network, indirectly leading to the abnormal enrichment of H3K4me3 (39, 40). Under normal physiological conditions, the level of H3K4me3 is regulated by the dynamic balance between “methyltransferases” and “demethylases”. However, the MLL/AF4 fusion protein can disrupt this balance by inhibiting the expression or activity of demethylases such as KDM5A. Meanwhile, it can also indirectly inhibit the clearance mechanism of H3K4me3 by regulating the expression of non-coding RNAs (e.g., miR-125b) (41). This dual effect of “promoting methylation” and “inhibiting demethylation” ultimately results in a significant increase in the level of H3K4me3 in the promoter regions of genes such as BCL2 and MYC.

In addition, although high methylation of the ABCB1 promoter in MLL^+^ children inhibits its mRNA expression (42), decitabine can restore ABCB1 expression through demethylation and enhance the sensitivity of leukemia cells to chemotherapy drugs (decreased IC₅₀) (43). This provides experimental evidence for the clinical combined use of demethylating drugs.

High methylation of the TP53 promoter in E2A/PBX1^+^ children suggests that epigenetic silencing of tumor suppressor genes may be one of the reasons for their poor prognosis (44). Drugs targeting DNA methyltransferases may reverse this process. In conclusion, abnormal epigenetic modifications are important synergistic mechanisms driven by fusion genes in leukemia occurrence. Detecting their status can provide new targets for individualized treatment (such as combined epigenetic drugs).

### 4.2 Association between molecular genetic characteristics and clinical phenotypes

**Figure 9:**
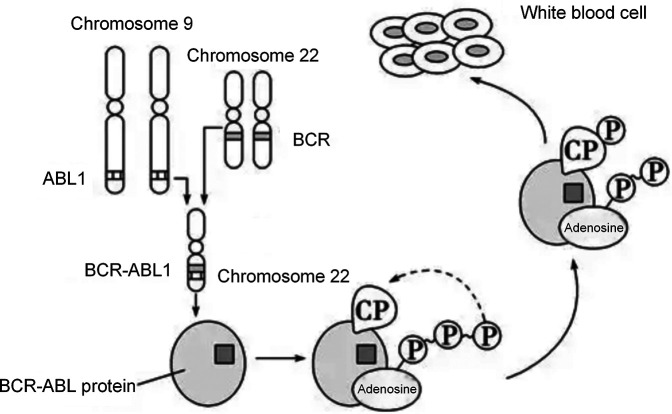
The process of BCR—ABL fusion gene formation and the activation of tyrosine kinase pathway driving leukemia.

This study found that there were significant differences in white blood cell counts (WBC) among children with positivity for different fusion genes. Children with BCR/ABL positivity had the highest WBC level, while those with ETV6/RUNX1 positivity had the lowest. Moreover, 73.7% of BCR/ABL-positive children had WBC > 100 × 10⁹ L^−^¹, which is closely related to the high carcinogenicity of this gene. The tyrosine kinase encoded by the BCR/ABL fusion gene can continuously activate downstream signaling pathways (such as RAS, PI3K/AKT) (45), leading to abnormal proliferation of leukemia cells and thus a significant increase in peripheral blood white blood cell counts. Figure 9 depicts the intricate process of the formation of the BCR—ABL fusion gene and its subsequent activation of the tyrosine kinase pathway, which is a crucial mechanism underlying leukemia development. This result provides a basis for clinically preliminary prediction of high-risk genes through WBC levels.

In addition, the association analysis between immunophenotyping and fusion genes showed that E2A/PBX1 accounted for as high as 72.73% in pre-B-ALL, while ETV6/RUNX1 and BCR/ABL were mainly distributed in C-B-ALL. This distribution pattern is related to the molecular regulatory mechanisms at the stage of cell differentiation. B cells in the pre-B-ALL stage have not completed immunoglobulin heavy chain rearrangement (46), and the E2A/PBX1 fusion gene may block B cell differentiation at the pre-B stage by inhibiting the transcriptional activity of E2A (47). As a more mature B cell subtype, the pathogenesis of C-B-ALL may be related to abnormal differentiation of hematopoietic stem cells mediated by ETV6/RUNX1.

### 4.3 Analysis of consistency between fusion genes and chromosomal abnormalities

Chromosomal translocation is the main mechanism for fusion gene formation. However, this study found significant differences in the consistency between different genes and chromosomal abnormalities. The consistency of ETV6/RUNX1 and BCR/ABL with their corresponding chromosomal translocations [*t*(12; 21) and *t*(9; 22)] was 100%, while that of E2A/PBX1 and MLL with their corresponding abnormalities [*t*(1; 19) and 11q23 changes] was only 47.1%. This result reveals the diversity of fusion gene formation mechanisms.

The fusion of ETV6/RUNX1 and BCR/ABL is completely dependent on specific chromosomal translocations (48). The *t*(12; 21) translocation involves the exchange between the short arm of chromosome 12 and the long arm of chromosome 21, which can stably produce the ETV6/RUNX1 fusion gene. Thus, its consistency with chromosomal abnormalities is extremely high (49). In this study, all 44 ETV6/RUNX1-positive children were detected with *t*(12; 21), among whom 6 cases were combined with other chromosomal abnormalities (such as deletion of chromosome 9). This suggests that these non-specific abnormalities may enhance the malignant phenotype of leukemia through synergistic effects.

The formation of E2A/PBX1 and MLL fusion genes may involve internal gene rearrangement or abnormal epigenetic regulation in addition to chromosomal translocation (50). For example, 9 E2A/PBX1-positive children in this study had normal karyotypes but positive gene detection results. This may be caused by fusion due to chromosomal microtranslocation or retrotransposition, which needs further verification by techniques such as fluorescence *in situ* hybridization. The MLL gene is located in the 11q23 region and easily fuses with multiple partner genes (such as AF4, AF9) (51). Some fusions may be caused by chromosomal insertion rather than translocation, making them difficult to detect by karyotype analysis. Hence, the consistency with 11q23 abnormalities is low.

### 4.4 Rarity and literature support of multi-fusion gene events

The coexistence of multiple common fusion genes (2 cases with double fusions and 1 case with triple fusions) observed in our 302-child cohort is indeed a rare molecular event in pediatric acute lymphoblastic leukemia (ALL), as most classic driver genetic abnormalities in ALL are considered mutually exclusive in traditional 认知. However, accumulating clinical studies have documented similar multi-fusion cases, providing robust evidence for the biological plausibility of such phenomena and supporting the authenticity of our findings.

Early evidence came from Balatzenko et al. (2013), who reported a 3-year-old boy with B-cell ALL (B-ALL) co-positive for ETV6-RUNX1 and BCR-ABL1 (e1a2, P190 subtype) fusion transcripts (52). Clinically, the child presented with fever, diarrhea, normal white blood cell count (5.9 × 10⁹/L) without circulating abnormal cells, anemia, thrombocytopenia, and hepatomegaly; bone marrow examination showed hypercellularity with agranular lymphoid blast cells expressing pre-B immunophenotype (cyCD79α^+^CD19^+^sCD22^+^CD10^+^CD34^+^sIgM^−^) and dim myeloid-associated markers (CD13^+^CD33^+^). Although conventional cytogenetic analysis failed to yield results, molecular detection confirmed the co-occurrence of the two fusions. The patient was treated with the AIEOP-BFM-ALL2000 protocol, achieved complete remission after the first induction course, and maintained residual disease <0.05% at the 12-month follow-up. This case closely parallels our Case 3 (ETV6/RUNX1 + BCR/ABL + MLL/ENL) in terms of “low-risk + high-risk fusion coexistence”—both involve ETV6-RUNX1 (a classic low-risk marker) and BCR-ABL1 (a high-risk driver)—and verifies that such multi-fusion clones can respond to standardized chemotherapy, laying a foundation for understanding the clinical management of similar cases in our study.

Subsequent studies have further expanded the spectrum of multi-fusion subtypes. Barbosa et al. (2023) reported the second pediatric case of B-cell precursor ALL (BCP-ALL) with rare concomitance of ETV6::RUNX1 and BCR::ABL1p210 (P210 subtype) (53). The patient also harbored ETV6 deletion and copy number alterations (CNAs) compatible with the “IKZF1-plus” profile—a genetic feature often associated with poor prognosis. Notably, the authors emphasized that ETV6::RUNX1 and BCR::ABL1 are both founder leukemogenic events in pediatric BCP-ALL (accounting for 20%–25% and 2%–3% of cases, respectively), and their coexistence had only been reported in one pediatric and one young adult case before this study. This rarity aligns with our observation of only 3 multi-fusion cases in 302 children, while the association between additional genetic alterations (e.g., ETV6 deletion, IKZF1 abnormalities) and multi-fusion phenotypes also echoes our finding that Case 3 (triple fusion) had high-risk clinical features (poor MRD clearance), suggesting that multi-fusion events may synergize with other genetic aberrations to exacerbate disease malignancy.

In relapsed ALL, multi-fusion events may also be underrecognized due to limitations of initial diagnostic algorithms. Wu et al. (53) described a child with B-ALL who experienced two relapses with short event-free intervals; cytogenetic analysis at diagnosis identified the *t*(12; 21) translocation causing ETV6::RUNX1 fusion, but molecular genetic testing at the second relapse revealed a concurrent interstitial deletion of chromosome X leading to P2RY8::CRLF2 fusion (54). As ETV6::RUNX1 and P2RY8::CRLF2 each define distinct molecular subclasses of B-ALL with unique mutational landscapes and prognoses, the authors highlighted that co-existing leukemogenic aberrations may significantly modify treatment response and relapse risk but are often missed in initial workups relying solely on standard cytogenetics. This finding underscores the importance of our study's comprehensive molecular detection strategy (combining qPCR, WES, FISH, and Sanger sequencing), which enabled us to identify multi-fusion cases at diagnosis rather than during relapse, providing a basis for early risk stratification and intensive treatment.

Collectively, these studies confirm that while multi-fusion events (double/triple fusions) are rare in pediatric ALL, they are biologically and clinically relevant phenomena rather than technical artifacts. The consistency between our cases and the reported literature—including the types of co-existing fusions (e.g., ETV6::RUNX1 + BCR::ABL1), association with high-risk genetic features, and clinical responsiveness to intensive treatment—further validates the reliability of our findings. More importantly, these cases collectively suggest that multi-fusion events may arise from secondary chromosomal translocations in leukemia clones during disease progression or relapse, and their detection requires a combination of multi-platform molecular technologies to avoid underdiagnosis. For clinical practice, such cases should be classified as very high-risk, and individualized treatment regimens (e.g., combining tyrosine kinase inhibitors for BCR::ABL1 and demethylating drugs for MLL fusions) should be developed to improve outcomes.

### 4.5 Impact of molecular genetic characteristics on treatment response and prognosis

Prognostic analysis in this study showed significant differences in treatment outcomes among children with different fusion genes. Children with ETV6/RUNX1 positivity had the highest complete remission rate, the lowest recurrence rate, and a 2-year event-free survival rate of 89.8%. In contrast, children with BCR/ABL positivity had a complete remission rate of only 57.9% and a 2-year event-free survival rate of 42.1%. These differences are directly related to the biological characteristics of the genes themselves.

The ETV6/RUNX1 fusion gene affects hematopoietic differentiation by inhibiting the transcriptional activity of RUNX1, but it is highly sensitive to chemotherapy drugs (such as glucocorticoids and vincristine), leading to a favorable prognosis. BCR/ABL, however, continuously activates tyrosine kinase signaling, which can cause cellular resistance to chemotherapy (55). In this study, BCR/ABL-positive children with WBC > 100 × 10⁹ L^−^¹ had a 2-year event-free survival rate of only 28.6%, suggesting that high white blood cell count may further worsen the prognosis. This supports the necessity of early combined treatment with tyrosine kinase inhibitors for such children.

Children with MLL positivity had poor prognosis. The mechanism is related to MLL fusion proteins recruiting epigenetic modification enzymes, leading to disordered gene expression (56). Infant ALL with MLL rearrangement responds poorly to conventional chemotherapy, requiring the exploration of more intensive treatment regimens (such as hematopoietic stem cell transplantation). The prognosis of E2A/PBX1-positive children is between the above two groups, which may be related to their moderate sensitivity to drugs such as cyclophosphamide (57).

This study analyzed the association between fusion genes and dynamic changes in MRD. It found that gene type is a key factor affecting the speed of MRD clearance. ETV6/RUNX1^+^ children had the fastest MRD clearance, with a negativity rate of 86.4% on day 33, consistent with the high sensitivity of this gene to chemotherapy. In contrast, BCR/ABL^+^, MLL^+^, and Ph-like ALL children had a high rate of persistent MRD positivity, reflecting the resistance of their leukemia cells to conventional chemotherapy.

Gene co-mutations further delay MRD clearance. For example, BCR/ABL^+^ children with concurrent FLT3/ITD mutations all showed persistent MRD positivity, suggesting that multi-target combined therapy may be more effective. In addition, positive MRD on day 33 is a strong predictor of poor prognosis, especially in BCR/ABL^+^ children. The impact of MRD status on survival even exceeds that of gene type itself, indicating that dynamic MRD monitoring can make up for the limitations of single gene detection in risk stratification. The association analysis between fusion gene type and dynamic changes in MRD can combine static gene characteristics with dynamic treatment responses, more accurately identify high-risk children, and provide real-time basis for individualized treatment adjustment.

This study still has certain limitations. In terms of sample size, although the 302 newly diagnosed children cover major immune subtypes and age groups, the number of detected cases of some rare fusion genes (such as NUP214/ABL1) and Ph-like ALL-related molecules (such as SH2B3 deletion with only 3 cases) is small. This may lead to insufficient statistical power and make it difficult to fully reveal their clinical associations. Therefore, expanding the sample size for further verification is needed in the future. Secondly, there are limitations in detection technologies. In chromosome karyotype analysis, some children have insufficient bone marrow cell metaphases (such as 15 cases of ETV6/RUNX1-positive children with no metaphases), which may miss potential chromosomal abnormalities. Although high-throughput whole-exome sequencing covers common and rare fusion genes, it does not include comprehensive analysis of structural variations. Moreover, its sensitivity in detecting low-frequency mutations (allele frequency <5%) is limited, which may affect the integrity of molecular genetic characteristics.

The follow-up time is relatively short. This study mainly analyzes the 2-year event-free survival rate, while the association between long-term prognosis of childhood ALL (such as 5-year survival rate and long-term complications) and molecular genetic characteristics remains unclear. It is necessary to extend the follow-up period to improve the prognosis evaluation system. In addition, epigenetic modification analysis only focuses on limited subtypes such as MLL^+^ and E2A/PBX1^+^, and does not cover Ph-like ALL and other rare gene-positive children. The universality of their epigenetic regulatory mechanisms needs further exploration.

In *in vitro* experiments, the inhibitory effect of decitabine on MLL^+^ cells is only based on short-term culture (72 h). There is a lack of verification in *in vivo* animal models, and its practical application value in clinical treatment needs further confirmation through clinical trials.

### 4.6 Potential applications in precision medicine

Traditional risk stratification for childhood ALL mostly relies on clinical indicators such as age and initial WBC count. It has limitations including strong subjectivity and insufficient accuracy. The molecular genetic characteristics revealed in this study can serve as core supplementary indicators. They help build a two-dimensional stratification model combining clinical indicators and molecular markers, significantly improving the accuracy of risk stratification.

Clinically, children positive for the ETV6/RUNX1 fusion gene without other high-risk co-mutations can be clearly classified into the low-risk group to avoid over-treatment. For these children, the dosage of chemotherapy drugs can be appropriately reduced. This ensures therapeutic efficacy while lowering the risk of long-term complications. Children positive for BCR/ABL, with MLL rearrangement, or with Ph-like ALL are typical high-risk groups. They have the lowest complete remission rates. Based on the data of this study, clinical practice can take the positivity of such molecular markers as the core basis for high-risk stratification. Even for subtypes with worse prognosis—such as BCR/ABL combined with FLT3/ITD co-mutation, or MLL/AF4 rearrangement—they can be further classified into the “very high-risk” category. This provides a basis for subsequent intensive treatment.

Children with different molecular genetic subtypes of ALL show significant differences in sensitivity to therapeutic drugs. The data of this study can directly guide clinical practice in selecting targeted treatment plans. This improves therapeutic efficacy and reduces the occurrence of drug resistance. Regarding the drug sensitivity characteristics of specific molecular subtypes, this study provides clear indications for targeted therapy. For example, children positive for BCR/ABL (especially the P190 and P210 subtypes) can receive early combination therapy with tyrosine kinase inhibitors. This makes up for the defect of drug resistance in traditional chemotherapy. For the CRLF2 rearrangement subtype in Ph-like ALL, its pathogenesis is related to the abnormal activation of the JAK-STAT pathway. The data of this study can support the clinical trial of JAK inhibitors. This improves the poor early prednisone response in 71.4% of children with this subtype. Children with MLL rearrangement have high methylation in the promoter region of the ABCB1 gene. *in vitro* experiments have confirmed that decitabine can restore ABCB1 expression through demethylation and reduce the IC₅₀. Therefore, clinical practice can explore the combined regimen of decitabine and chemotherapy to reverse chemotherapy resistance in children positive for MLL.

Based on the data on the correlation between MRD and molecular subtypes, clinical practice can dynamically optimize chemotherapy intensity. Children positive for ETV6/RUNX1 show rapid MRD clearance. After MRD turns negative on day 33 of induction remission, the course of consolidation therapy can be appropriately shortened or the drug intensity can be reduced. However, even if children with MLL rearrangement or BCR/ABL positivity achieve initial remission, they still need prolonged consolidation therapy or increased drug dosage. Especially for BCR/ABL-positive children with positive MRD on day 33, more aggressive treatment methods (such as hematopoietic stem cell transplantation) should be considered.

The data of this study provides references for the key time points and early warning thresholds of MRD monitoring. The MRD status on day 15 and day 33 of induction remission has important prognostic value. The MRD positive rates of children with BCR/ABL positivity or MLL rearrangement reach 73.68% and 72.00% respectively on day 15. These children require close monitoring of subsequent MRD changes. Children with positive MRD on day 33 have a 2-year recurrence rate of 41.2%, which is significantly higher than the 5.8% of children with negative MRD. Therefore, clinically, MRD ≥ 10^−^^4^ on day 33 can be used as an early warning threshold for high recurrence risk, and treatment plans should be adjusted in a timely manner.

Rare subtypes identified in this study—such as ZNF384 rearrangement and DUX4 rearrangement—have low incidence rates. However, they have unique associations with immunophenotypes (e.g., 85.7% of ZNF384 rearrangements are concentrated in pre-B-ALL) and potential pathogenic mechanisms (e.g., ZNF384 regulates PAX5 to affect B-cell differentiation). They can serve as entry points for the development of new targets. The characteristic that the NUP214/ABL1 fusion subtype is sensitive to imatinib suggests that ABL inhibitors may cover more atypical ABL fusion subtypes. This provides a basis for expanding the application scope of targeted therapy.

## 5 Conclusion

This study systematically analyzed the molecular genetic characteristics and clinical data of 302 newly diagnosed children with ALL. The molecular genetic characteristics of childhood ALL show significant diversity and clinical significance. The total positive rate of fusion genes is 50.66%. The gene detection rate in B-ALL children (52.90%) is significantly higher than that in T-ALL (37.21%). Common fusion genes such as ETV6/RUNX1 (19.54%), MLL (9.27%), and BCR/ABL (6.29%) are the main ones. A certain proportion of rare fusion genes (4.97%) and Ph-like ALL-related molecular abnormalities (9.27%) are also detected. These molecular genetic characteristics have specific distributions in terms of age and immune subtypes.

The consistency between fusion genes and chromosomal abnormalities varies. ETV6/RUNX1 and BCR/ABL show 100% consistency with specific chromosomal translocations *t*(12; 21) and *t*(9; 22) respectively. However, the consistency between E2A/PBX1, MLL and their corresponding chromosomal abnormalities is about 50%, suggesting that the formation mechanisms of different fusion genes are different.

Molecular genetic characteristics are closely related to clinical treatment response and prognosis. Children with ETV6/RUNX1 positivity have the highest complete remission rate (93.2%) and the best 2-year event-free survival rate (89.8%), with a good prognosis. Children with BCR/ABL positivity have the lowest complete remission rate (57.9%) and the worst 2-year event-free survival rate (42.1%), with a poor prognosis. The prognosis of children with MLL or E2A/PBX1 positivity is between the above two groups.

In addition, the dynamic changes of MRD are closely related to gene types. The MRD positive rate is significantly higher in children with high-risk genes, and MRD status can be used as an important indicator for evaluating prognosis. At the same time, some children with positive fusion genes have abnormal epigenetic modifications, which are related to chemotherapy sensitivity and gene expression, providing experimental basis for combined epigenetic drug therapy.

In summary, the molecular genetic characteristics of childhood ALL have important value in disease classification, risk stratification, treatment option selection and prognosis evaluation. The combination of fusion gene detection and dynamic MRD monitoring can provide key basis for the accurate diagnosis and individualized treatment of childhood ALL, and help to further improve the overall efficacy and long-term prognosis of childhood ALL.

## Data Availability

The original contributions presented in the study are included in the article/supplementary material, further inquiries can be directed to the corresponding author.
